# Altered metabolome and microbiome features provide clues in understanding irritable bowel syndrome and depression comorbidity

**DOI:** 10.1038/s41396-021-01123-5

**Published:** 2021-11-08

**Authors:** Lijuan Han, Ling Zhao, Yong Zhou, Chao Yang, Teng Xiong, Lin Lu, Yusheng Deng, Wen Luo, Yang Chen, Qinwei Qiu, Xiaoxiao Shang, Li Huang, Zongchao Mo, Shaogang Huang, Suiping Huang, Zhi Liu, Wei Yang, Lixiang Zhai, Ziwan Ning, Chengyuan Lin, Tao Huang, Chungwah Cheng, Linda L. D. Zhong, Shuaicheng Li, Zhaoxiang Bian, Xiaodong Fang

**Affiliations:** 1 State Key Laboratory of Dampness Syndrome of Chinese Medicine, The Second Affiliated Hospital of Guangzhou University of Chinese Medicine, Guangzhou, China; 2 KMHD, Shenzhen, China; 3 Institute of Brain and Gut Research, School of Chinese Medicine, Hong Kong Baptist University, Hong Kong SAR, China; 4 Academy of Integrative Medicine, Shanghai University of Traditional Chinese Medicine, Shanghai, China; 5 Department of Computer Science, City University of Hong Kong, Hong Kong SAR, China; 6 Department of Computer Science, Hong Kong Baptist University, Hong Kong SAR, China; 7 Department of Neurology, The First Affiliated Hospital of Guangzhou Medical University, Guangzhou, China; 8 School of Biology and Biological Engineering, South China University of Technology, Guangzhou, China; 9 Department of Biotechnology, Huazhong University of Science and Technology, Wuhan, China; 10 Department of Biomedical Engineering, City University of Hong Kong, Hong Kong SAR, China; 11 BGI-Shenzhen, Shenzhen, China; 12 China National GeneBank, BGI-Shenzhen, Shenzhen, China

**Keywords:** Clinical microbiology, Colitis, Metagenomics

## Abstract

Irritable bowel syndrome (IBS) is one of the functional gastrointestinal disorders characterized by chronic and/or recurrent symptoms of abdominal pain and irregular defecation. Changed gut microbiota has been proposed to mediate IBS; however, contradictory results exist, and IBS-specific microbiota, metabolites, and their interactions remain poorly understood. To address this issue, we performed metabolomic and metagenomic profiling of stool and serum samples based on discovery (*n* = 330) and validation (*n* = 101) cohorts. Fecal metagenomic data showed moderate dysbiosis compared with other diseases, in contrast, serum metabolites showed significant differences with greater power to distinguish IBS patients from healthy controls. Specifically, 726 differentially abundant serum metabolites were identified, including a cluster of fatty acyl-CoAs enriched in IBS. We further identified 522 robust associations between differentially abundant gut bacteria and fecal metabolites, of which three species including *Odoribacter splanchnicus*, *Escherichia coli*, and *Ruminococcus gnavus* were strongly associated with the low abundance of dihydropteroic acid. Moreover, dysregulated tryptophan/serotonin metabolism was found to be correlated with the severity of IBS depression in both fecal and serum metabolomes, characterized by a shift in tryptophan metabolism towards kynurenine production. Collectively, our study revealed serum/fecal metabolome alterations and their relationship with gut microbiome, highlighted the massive alterations of serum metabolites, which empower to recognize IBS patients, suggested potential roles of metabolic dysregulation in IBS pathogenesis, and offered new clues to understand IBS depression comorbidity. Our study provided a valuable resource for future studies, and would facilitate potential clinical applications of IBS featured microbiota and/or metabolites.

## Introduction

Irritable bowel syndrome (IBS) is a common functional gastrointestinal disorder that affects around 11% of the population globally [1]. IBS is categorized into four subtypes including diarrhea-predominant IBS (IBS-D), constipation-predominant IBS (IBS-C), mixed IBS (IBS-M), and un-subtyped IBS (IBS-U) according to patients’ bowel habits. Moreover, psychological comorbidity including anxiety and depression is common in IBS population [2]. Although the precise mechanism of IBS remains unclear, the alteration of gut microbiota has been proposed to mediate IBS pathogenesis.

Pittayanon and colleagues systematically reviewed gut microbiota studies in IBS from inception to April, 2018, reported the IBS-specific intestinal microbiota characterized by an increase in family *Enterobacteriaceae*, family *Lactobacillaceae* and genus *Bacteroides*, together with a decrease in uncultured *Clostridiales* I, genus *Faecalibacterium* and genus *Bifidobacterium* [3]. However, inconsistent results exist among studies, probably due to the differences in methodology, the limited sample size, coupled with the lack of necessary information such as antibiotics/probiotics use and diet habit. For example, one study showed a relative increased abundance of bacterial species including *Enterobacteriaceae* in feces of IBS patients, with decreased composition in *Lactobacillus* and *Bifidobacterium*; while opposite results found in another study described an increase in *Lactobacillus* genus or *Lactobacillales* in IBS-D [4]. Tap et al. reported the fecal and mucosal microbiota signatures that are related with the severity of IBS [5]. Vich et al*.* reported a large scale of metagenomic sequencing of stool samples from 1792 individuals with IBS and inflammatory bowel disease (IBD), which demonstrate overlapped and specific species between IBD and IBS patients compared with controls [6]. Jeffery et al*.* reported significant differences in fecal microbiomes and metabolomes of individuals with or without IBS, and highlighted the potential of using metabolomes and microbiome in the diagnosis and treatment of IBS [7]. However, by analyzing the fecal and mucosa-associated microbiome of IBS patients from a Swedish random population, it was reported that no IBS-specific microbiota feature was identified [8].

The microbial composition can shape the environment in the colon as metabolites produced from microbes can be involved in signaling, immune system modulation, or have antibiotic activity. For example, short-chain fatty acids (SCFAs) showed lower proportions in sera of IBS patients [9], which play major roles in the microbiota−host interaction by inhibiting inflammatory and malignant growth [10]. A recent study from our group revealed an impaired bile acid synthetic regulation with excessive bile acid excretion contributed by the *Clostridia*-rich microbiota that is associated with the severity of diarrheal symptoms in IBS-D patients [11].

These results indicate a potential role of microbiota and microbiota-related byproducts in IBS, but the alterations of fecal and serum metabolites and their interactions with gut microbiota are not well established and interpreted. Here in this study, we integrated microbiome and untargeted metabolites profiling data from discovery (*n* = 330) and validation (*n* = 101) cohorts to understand the IBS-specific microbiota and metabolite alterations. We found a moderate alteration of fecal microbiome and metabolome in IBS patients, whereas clear separation between IBS patients and healthy controls was observed using serum metabolites. Interestingly, we found strong associations between fecal metabolites and microbiota, and identified dysregulated tryptophan/serotonin metabolism correlated with the severity of IBS depression. Collectively, our study provides a valuable resource to understand IBS-specific microbiota/microbiome features and interactions, highlights the potential application of serum metabolites in laboratory diagnostics of IBS, and offers new clues to understand IBS depression comorbidity.

## Materials and methods

### Sample collection

Adults meeting the Rome IV criteria for IBS [12, 13] were prospectively recruited at two Chinese medicine clinics affiliated with the School of Chinese Medicine, Hong Kong Baptist University. More specifically, IBS patients were recruited on if they fulfilled the following inclusion criteria: (1) meeting the Rome IV criteria, including recurrent abdominal pain on average at least 1d/week in the last 3 months; (2) IBS Symptom Severity Scale (IBS-SSS) over than 75 points at baseline; (3) age of 18−65 years; (4) normal colonic evaluation with 5 years by examination of colonoscopy or barium enema; (5) Written informed consent. Patients were excluded if they fulfilled one or more of the following exclusion criteria: (1) pregnancy or breast-feeding; (2) medical history of IBD, carbohydrate malabsorption, hormonal disorder, known allergies to food additives, and/or any other serious diseases; (3) surgical histories of gallbladder removal, gastrointestinal (GI) tract, and cerebral cranium; (4) having parasitic infections; (5) use of medications known to influence gastrointestinal function, blood pressure, and fat. The disease status was classified by predominant bowel habits on the days with abnormal bowel movements based on the questionnaire of Bristol Stool Form scale and defecation frequency [12]. Matched healthy controls without the medical history of neurodegenerative diseases, cardiovascular diseases, metabolic disorders, GI diseases, and surgical histories of gallbladder removal, GI tract, and cerebral cranium were also recruited in the same clinical centers. Included subjects were instructed to provide morning first stool samples and fasting blood samples on the same day for biochemical detection and omics analyses. All included participants were required to stop using antibiotics, probiotics, prebiotics, and other microbiota-related supplements at least three weeks before stool sampling. Specimens (serum and stools) were transported to the laboratory using dry ice and were frozen at −80 °C.

In total, we recruited 330 and 101 individuals for discovery and validation cohorts, respectively. Specifically, we collected 330 fecal samples (including 24 IBS-C, 214 IBS-D, 19 IBS-M, 7 IBS-U, and 66 healthy controls) and 325 matched serum samples (including 24 IBS-C, 209 IBS-D, 19 IBS-M, 7 IBS-U, and 66 healthy controls) from the discovery cohort, and collected 101 fecal samples (including 7 IBS-C, 57 IBS-D, 13 IBS-M, 9 IBS-U, and 15 healthy controls) and 98 matched serum samples (including 7 IBS-C, 55 IBS-D, 13 IBS-M, 9 IBS-U, and 14 healthy controls) from the validation cohort.

There were 24 intrinsic factors and 45 questionnaire factors collected for each subject. The 24 intrinsic factors contained: Gender, Age, BMI, Subtype (including IBS-D, IBS-C, IBS-M, and IBS-U), Serum TBA (Serum Total Bile Acids (TBAs), umol/g), Fecal TBA (Fecal TBAs, umol/g), ALP (Alkaline phosphatase, u/L), ALT (Alanine transaminase, u/L), AST(Aspartate transaminase, u/L), Urea (mmol/L), Creatinine (umol/L), TC (Serum total cholesterol, mmol/L), Fasting glucose (mmol/L), TG (Triglyceride, mmol/L), C4 (serum 7α-hydroxy-4-cholesten-3-one, ng/mL), FGF19 (serum fibroblast growth factor 19, pg/mL), Stool Freq (stool frequency per day), Bristol Stool Score, HAMD (Hamilton Depression Rating Scale), SAS (the Zung Self-Rating Anxiety Scale), SDS (the Zung Self-Rating Depression Scale), IBS SSS Pain (IBS Symptom Severity Scale-Pain Score), IBS SSS Distention (IBS Symptom Severity Scale-Distention Score) and IBS SSS (IBS Symptom Severity Scale). The 45 questionnaire factors contained: (1) basic information (Education Level, Marriage and Monthly Income); (2) dietary habits (Rice Products Quantity and Frequency, Flour Products Quantity and Frequency, Coarse Cereals Quantity and Frequency, Viscera Blood Products Quantity and Frequency, Red Meat Quantity and Frequency, White Meat Quantity and Frequency, Egg Products Quantity and Frequency, Vegetables Quantity and Frequency, Tuber Products Quantity and Frequency, Bean Products Quantity and Frequency, Fruit Quantity and Frequency, Vegetable Oil Quantity and Frequency, Animal Oil Quantity and Frequency); (3) beverage consumption (Tea Quantity and Frequency, Coffee Quantity and Frequency, Alcohols Quantity and Frequency, Milk Products Quantity and Frequency); (4) physical exercise habits (Physical Activity Quantity, Exercise Quantity and Frequency); (5) sleeping status (Sleep Quality and Sleep Time); (6) smoking (Smoke Duration); (7) stress status (Spirit Quantity and Frequency).

### Depression diagnosis

IBS patients with depression were diagnosed by a combination of a SDS self-report and a 17-item HAMD-17 other-report. All subjects were firstly requested to complete the SDS, with an index score of 53 as a cut-off value [14]. Those with SDS score greater than 53 were received a professional diagnosis with a HAMD by a psychiatrist. The severity of depressive symptom was categorized by the following severity range [15]: (1) HAMD < 7: no depression (rIBS); (2) HAMD 8−16: mild depression (mIBS); (3) HAMD ≥ 17: moderate and severe depression (17−23 for moderate and ≥24 for severe) (sIBS). In the 330-member discovery cohort, 264 IBS patients were further diagnosed as 185 rIBS, 63 mIBS, and 16 sIBS.

### Approval for human patient research

This study was approved by the Ethics Committee on the Use of Human & Animal Subjects in Teaching & Research (Approval no. HASC/15-16/0300 and HASC/16-17/0027). Written informed consent was obtained from each participant prior to sample collection.

### Measurements of total bile acids, 7α-hydroxy-4-cholesten-3-one and fibroblast growth factor 19 in serum samples

Concentrations of TBAs and fibroblast growth factor 19 (FGF19) in human serum were tested by using commercial TBA Assay Kit (Cell Biolabs, San Diego, CA, USA) and Human Fibroblast growth Factor 19 Assay Kit (Thermo Scientific, Waltham, MA, USA), respectively. The level of serum 7α-hydroxy-4-cholesten-3-one (C4) was quantified by a liquid chromatography coupled with mass spectrometry (LC/MS)-based method developed from our group.

### Metabolite profiling of fecal and serum samples

Metabolites were extracted from serum and fecal samples as described previously [16, 17]. Briefly, feces (100 mg) were completely homogenized with five-fold volume of ice-cold distilled water. After high-speed centrifugation (13,000 rpm for 15 min at 4 °C), water extractions were transferred to a new 2 mL tube. Subsequently, another five-fold volume (500 μL) of methanol was added into the pellet sample. The mixture was completely homogenized and centrifuged again. Methanol extractions were combined with the previous water extractions. Serum (50 μL) was prepared with four volumes of cold methanol for protein precipitation, and metabolite extracts were obtained after vortex and centrifugation. The 200 μL of fecal or serum supernatant was dried and redissolved in the same volume of solvent consisting of water and acetonitrile (98:2, v/v). Meanwhile, quality control (QC) samples pooling all samples were individually prepared using the same protocol. P-chlorophenylalanine (5 μg/mL) was added as an internal standard.

### Analytical conditions for the untargeted metabolic profiling

The 2 μL of the resulting supernatant was injected into a liquid chromatography system (UPLC, Agilent 1290 Infinity, USA) and separated by gradient elution with 0.35 mL/min of flow rate using ACQUITY UPLC BEH C18 column (1.7 μm, 2.1 × 50 mm, Waters Corporation, Milford, MA). The gradient program consisted of phase A (0.1% formic acid in water) and phase B (0.1% formic acid in acetonitrile), which started from 2 to 5% B in 1 min, then raised to 100% B in the next 11 min and maintained at 100% B for 3 min, finally turned back to 2% B in 2 min. A quadruple time-of-flight mass spectrometer (Q-TOF/MS, Agilent 6543, USA) coupled with electrospray ionization (ESI) was performed for the acquisition of metabolic fragments in both positive and negative ionic modes. The instrument operated in full scan mode from 100 to 1,000 m/z and the capillary voltage was set at 3,000 V.

### Semi-quantification of neuroactive amides in feces and serum

A total of 17 neuroactive metabolites including tryptophan (TRP), tryptamine (TRPT), n-acetylserotonin (NAS), 5-hydroxyindoleacetic acid (5-HIAA), melatonin, kynurenine (KYN), kynurenic acid (KYA), serotonin, 3-indole acetic acid (3-IAA), 3-hydroxyanthranilic acid (3-HAA), tyrosine (TYR), succinic acid (SUCC), dopamine, glutamate (GLU), glutamine (GLN), histamine, aminobutyric acid (GABA) were purchased from Sigma-Aldrich (St. Louis, MO, USA). An isotopic glutamine-2,3,3,4,4-d5 as an internal standard was obtained from CDN isotopes (Pointe-Claire, Quebec, Canada). HPLC grade organic reagents for mass spectrometric analysis were purchased from Sigma-Aldrich (St. Louis, MO, USA). The standard curves and regression coefficients were gained based on internal standard adjustment.

The analytical conditions were referred to a published study [18]. Briefly, a liquid chromatography (Agilent UHPLC 1290, USA) coupled with a triple-quadrupole mass spectrometer (Agilent QQQ-MS 6438, USA) was applied. Sample injection and flow rate were set at 2 μL and 0.4 ml/min for each sample, respectively. Of neuroactive metabolites, GLU, GLN, histamine, GABA were separated using an ACQUITY BEH Amide column (1.7 μm, 100 mm × 2.1 mm) with a linear gradient of 100 mM ammonium formate in 95% water and 5% acetonitrile (mobile phase A) and 30 mM ammonium formate in 15% water and 85% acetonitrile (mobile phase B). The gradient program was: 100−80% B for the first 6 min, 80−50% B for 3 min, held at 50% B for 3 min, 50−100% B for 3 min. The column temperature was maintained at 30 °C. Moreover, melatonin, NAS, KYN, KYA, TRP, 5-HIAA, TYR, serotonin, 3-IAA, TRPT, 3-HAA, dopamine, SUCC were separated using an ACQUITY BEH C18 column (1.7 μm, 100 mm × 2.1 mm) with a linear gradient of 0.1% formic acid (FA) in water (mobile phase A) and 0.1% FA in acetonitrile (mobile phase B). The gradient program was: 2−30% B for the first 4 min, 30−100% B for 2 min, held at 100% B for 2 min, 100−2% B for 2 min. The column temperature was maintained at 40 °C. The capillary voltage of the mass spectrometer for both acquisitions was 3.5 kV in the positive mode. The acquisition data were analyzed using Agilent MassHunter Workstation Software for peak integration, calibration equations, and quantification of individual metabolites.

### Identification of differentially abundant metabolites

The nonparametric univariate method (Wilcoxon rank-sum test) was applied to identify metabolites that differed in abundance between IBS patients and controls and corrected for false discovery rate (FDR). Partial least-squares discriminant analysis (PLS-DA) was applied by using the function *plsda* from the R package *mixOmics*. Differentially abundant metabolites between IBS patients and healthy controls were identified by using the combined statistical criteria of PLS-DA Variable Importance in Projection (VIP) score > 1.0, FDR adjusted *p-*value < 0.1, and fold change in the range of 0.8−1.2.

### Unsupervised clustering of differentially abundant metabolites

We performed unsupervised clustering using residual abundance values from linear modelling described in detail by Fransoza et al. [32] on all differentially abundant metabolites. The detailed analysis process included two steps: calculating residual abundance values and clustering. To calculate residual abundance values, we firstly log-transformed the relative abundance values to variance-stabilize the data. Zero values were additively smoothed by half the smallest non-zero measurement on a per-sample basis. We then modelled the transformed abundance of each feature as a function of IBS phenotype (modelled as a categorical variable), with age as a continuous covariate. Residual abundance values from the linear models were used in subsequent cluster analyses.

We performed clustering on residual abundance values of all differentially abundant metabolites. This procedure enriches for covariation between metabolites. Metabolites were firstly ranked according to the richness in IBS (the fold change). The highest-ranked metabolite was assigned as an initial cluster. Each subsequent metabolite was then compared to each extant cluster. If the metabolite had a mean similarity to the cluster’s members exceeding a threshold, the metabolite was added to that cluster (using Spearman’s rank correlation *r* = 0.7 as a threshold). If the metabolite was not added to any extant clusters, it was seeded into a new cluster. After scanning all metabolites, clusters were renumbered according to their size, of which cluster 1 had the largest size, and so on.

### Metagenomic DNA extraction and sequencing

We used the phenol/chloroform/isoamyl alcohol method to extract microbial DNA from stool samples (200 mg) of included subjects [19]. The DNA that passed quality control was then subjected to library construction using the TruSeq DNA HT Sample Prep Kit. Paired-end sequencing (2 × 150 bp) was carried out using Illumina Hi-Seq platform.

### Read-level quality control and metagenomic profiling

Raw sequencing reads were filtered using SOAPnuke with the parameter “-l 20 -q 0.5 -n 0.1 -d” [20]. After the removal of adapter sequences and low-quality reads, host (human) reads were identified and removed by mapping against the human genome (hg19 build) with SOAP2 [21]. The remaining high-quality reads were used for further analysis. MetaPhlAn2 [22] (version 2.7.6) and default marker database mpa_v20_m200 were used to estimate the relative abundance of taxonomic profile. The database contained ~ 1 M clade-specific marker genes from approximately 17,000 reference genomes. We only considered the species-level data and kept species that exceed 0.1% relative abundance in at least six samples. Functional profiling was performed using HUMAnN2 [23] (version 0.9.4) in UniRef90 mode. Detected genes were further regrouped into gene families and pathways and then sum-normalized within HUMAnN2. Linear discriminant analysis Effect Size (LEfSe) [24] analysis was applied on the relative abundance of species, gene families, and pathways to identify disease-associated biomarkers. Features with Linear discriminant analysis (LDA) score > 2.0 and *p-*value < 0.05 were considered as statistically significant. Association between microbiome composition and covariates was estimated with PERMANOVA test using the R package *vegan*.

In order to compare gut microbiota profile of IBS with that of other diseases, the microbial taxonomic data including IBD [25], liver cirrhosis (LC) [26], colorectal cancer (CRC) [27], and type 2 diabetes (T2D) [28], were collected using the R package *curatedMetagenomicData* [29].

### Statistical analysis

We assessed which fraction of the total variation of Bray-Curtis distance of microbiome and metabolome can be explained by clinical factors using the function *adonis* from the R (3.5.1) package *vegan*. The *p-*value was determined by 1000x permutations and was further adjusted using the Benjamini and Hochberg method. The association between each factor and each diversity or richness measure was assessed by Spearman correlation. The 10-fold cross-validation random forest (RF) model was generated using R (3.5.1) package *randomForest*. The cross-validational error curves from 5 trials of the 10-fold cross-validation were averaged, and the minimum error in the averaged curve plus the standard error at that point was used as the cutoff. The minimum number of species/metabolites markers with an error less than the cutoff was chosen as the optimal model. The receiver operating characteristic (ROC) curves were drawn using R (3.5.1) package *pROC*, and area under the curve (AUC) scores are used to compare different models.

## Results

### Multi-omics profiles associated with intrinsic and questionnaire factors in IBS patients

To investigate the potential mechanism of pathogenesis in IBS, we collected 330 stool and 325 serum samples from a 330-member discovery cohort included 264 IBS patients and 66 healthy controls (Supplementary Fig. 1). Nearly 30% (79 out of 264) of IBS patients also suffered from depression or anxiety (Supplementary Table 1). For each subject, 24 intrinsic factors (including symptom scores and biochemical indices) and 45 questionnaire factors (including basic information, dietary habits, beverage consumption, exercise habits, sleeping status, smoking, stress status, etc) were collected, and the intercorrelations between factors were analyzed (Supplementary Fig. 2).

**Fig. 1:**
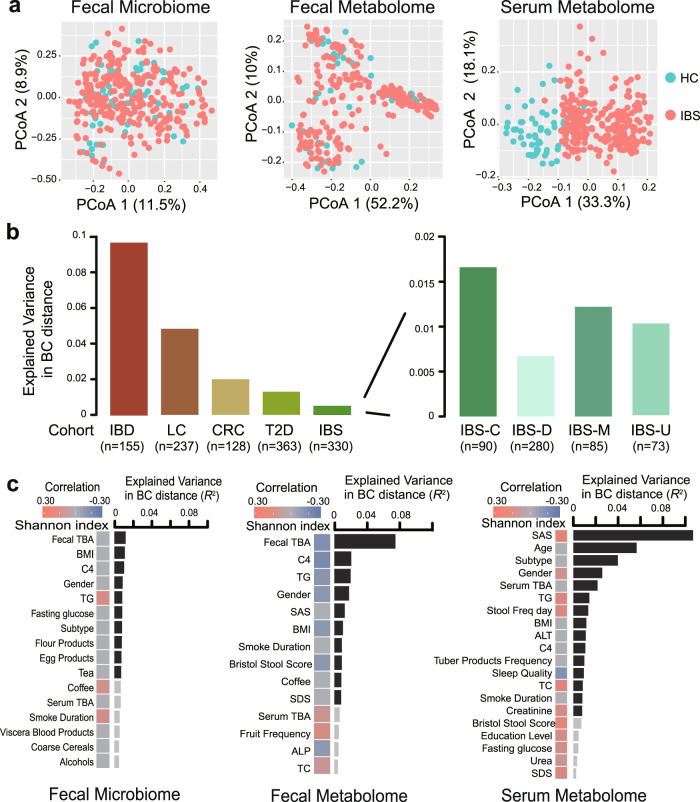
Multi-omics profiles of IBS patients. **a** Principal coordinates analysis (PCoA) of discovery cohort individuals based on 330 gut metagenomic (264 IBS and 66 HC), 330 fecal metabolomic (264 IBS and 66 HC), and 325 serum metabolomic profiles (259 IBS and 66 HC), respectively (Bray−Curtis distance). **b** Left panel shows the comparisons of gut microbiota dysbiosis in different diseases and different subtypes of IBS. The gut metagenomic data of other diseases, including inflammatory bowel disease (IBD, *n* = 155, including 121 IBD and 34 HC) [25], liver cirrhosis (LC, *n* = 237, including 123 LC and 114 HC) [26], colorectal cancer (CRC, *n* = 128, including 74 CRC and 54 HC) [27], and type 2 diabetes (T2D, *n* = 363, including 178 T2D and 185 HC) [28], were collected by the R package *curatedMetagenomicData* [29]. Right panel shows the comparisons of gut microbiota dysbiosis in different subtypes of IBS, including IBS-C (*n* = 90, 24 IBS-C and 66 HC), IBS-D (*n* = 280, 214 IBS-D, and 66 HC), IBS-M (*n* = 85, 19 IBS-M, and 66 HC), and IBS-U (*n* = 73, 7 IBS-U, and 66 HC). **c** Shown are associated factors with gut metagenomic, fecal metabolomic (negative ionization mode), and serum metabolomic (negative ionization mode) profiles in the 330-member discovery cohort. Black bars indicate statistical significance (FDR < 0.1).

To characterize the microbiome and metabolic profile in IBS, 330 stool samples were subjected to metagenomic sequencing and untargeted metabolomic analysis. In total, we captured 1788 known and uncharacterized fecal metabolites (including 856 identified in the positive mode and 932 identified in the negative mode). Regarding 325 serum samples available, we performed untargeted metabolomic analysis by using high-resolution mass spectrometer and captured 1769 known and uncharacterized serum metabolites (including 879 identified in the positive mode and 890 identified in the negative mode). We firstly assessed the overall differences between IBS patients and healthy controls in the fecal microbiome, fecal, and serum metabolomes respectively. Compared with healthy controls, IBS patients showed significantly elevated alpha and beta diversity in serum metabolome data (*p* = 1.04e−11 and 0.08 for alpha diversity by positive and negative ionization modes, respectively; *p* = 2.2e−16 and 2.2e−16 for beta diversity by positive and negative ionization modes, respectively; Supplementary Fig. 3a), increased beta diversity in fecal microbiome (*p* = 8.6e−09, Supplementary Fig. 3c), but no obvious differences in fecal metabolome data (Supplementary Fig. 3b). Interestingly, we noticed that the serum metabolites of IBS patients dramatically change from those of healthy controls, suggesting that serum metabolites have the potential to be used for IBS laboratory diagnostics (Fig. 1a). Such differences could result from a combination of multiple factors, including the effect of host disease status, the differences in dietary habits, medication use, etc. However, both IBS patients and healthy controls were clustered together on Principal coordinates analysis (PCoA) plot based on fecal metabolome and microbiome data, indicating fecal data have less power to distinguish IBS patients (Fig. 1a). We subsequently compared the degree of IBS-related microbiota dysbiosis to other microbiota-mediated diseases, including IBD, LC, CRC, and T2D. As shown in Fig. 1b, only 0.5% of the gut microbiota dysbiosis could be explained by disease status in IBS, which was similar to T2D but much smaller than that of LC or IBD, suggesting a moderate degree of microbiota dysbiosis in IBS compared to other diseases.

Further, we separately investigated the associations of intrinsic and questionnaire factors with metabolic and microbial profiles (Fig. 1c). Previous studies have suggested that the alterations of gut microbiome composition were influenced by clinical factors in IBD and IBS [6]. Here, we correlated those 24 intrinsic factors and 45 questionnaire factors with the overall composition of microbiota and metabolites based on Bray−Curtis dissimilarities, respectively. Permutational multivariate analysis of variance (PERMANOVA) revealed that IBS subtypes were associated with composition changes in both fecal microbiome and serum metabolome, but not with fecal metabolome. Based on fecal microbiome profiles, 10 factors were significantly associated (FDR < 0.1) with overall composition variation, which together explained 9% of inter-individual variations. The strongest associations with microbial composition were found to be the levels of several biochemical indices, including fecal TBA and C4, which is consistent with previous studies [30, 31]. Similar trends were observed in both positive and negative ionic mode fecal metabolome profiles (Fig. 1c and Supplementary Fig. 4a) which fecal TBA explained >6% of the composition variance that was far greater than any other factors. We also noticed that the fecal TBA was negatively correlated with the alpha diversity of fecal metabolomic profiling (Spearman’s *r* = −0.17, *p* = 0.002). By contrast, in serum metabolomics data, the Zung Self-Rating Anxiety Scale (SAS) primarily explained >8% of the compositional variance and showed a positive correlation with the Shannon index (Spearman’s *r* = 0.22, *p* = 0.0003) (Fig. 1c and Supplementary Fig. 4b). This result demonstrated a close linkage between psychological burden and serum metabolic alterations. Moreover, several dietary factors were also found to be associated with metabolomic profiles, of which the frequency of tea drinking showed a significant relationship with fecal metabolic variation (Spearman’s *r* = −0.14, *p* = 0.016) (Supplementary Fig. 4a, c).

### Metabolome alterations between IBS patients and healthy controls

To illuminate metabolic changes across groups, we performed PLS-DA on both serum and fecal metabolome data. The serum samples were largely separated between IBS and controls, which is consistent with broad changes in serum metabolite profiles described in earlier context (Supplementary Figs. 5b and 6b). By contrast, IBS patients could not be discriminated from healthy controls in fecal metabolome according to PLS-DA plots (Supplementary Fig. 7c, d). We then applied nonparametric univariate method (Wilcoxon rank-sum test) to identify differentially abundant serum metabolites between IBS patients and healthy controls. After correcting for FDR, a total of 726 serum metabolic fragments were significantly changed (FDR < 0.05) in IBS (Supplementary Figs. 5a and 6a and Supplementary Table 2), whereas only 8 fecal metabolites were identified (Supplementary Fig. 7a, b). When evaluating the generality of the differentially abundant serum metabolites in the validation cohort, 635 (87.5%) were enriched, of which 628 were also FDR significant (Supplementary Table 3).

**Fig. 2:**
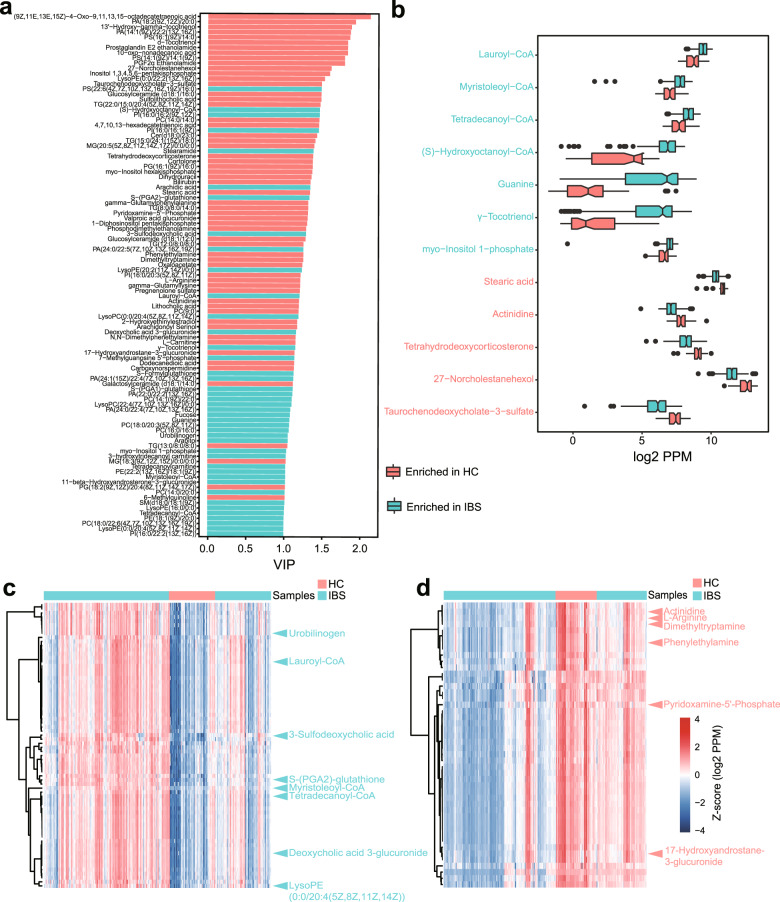
Differentially abundant serum metabolites and their clusters. By analyzing the serum metabolomic profiles of 259 IBS and 66 HC from the discovery cohort, we identified 726 differentially abundant serum metabolites (including 364 identified in the positive mode and 362 identified in the negative mode), among which 101 out of the 726 metabolites were structurally identified. **a** The barplot shows the PLS-DA VIP results in the projection of the 101 structurally identified differentially abundant serum metabolites. **b** Diet-associated differentially abundant serum metabolites that enriched in IBS patients and in healthy controls. The *Y*-axis represents each metabolite, and the *X*-axis shows the log-transformed PPM units of the abundance of each metabolite. **c** The largest cluster enriched in IBS contained 70 metabolites, and all of them significantly elevated among IBS patients. **d** One interesting cluster enriched in healthy controls contained 46 metabolites and included a variety of amines, all of which significantly elevated in healthy controls. Abundances are in log-transformed PPM units.

101 out of 726 different metabolic fragments in serum were structurally identified (Fig. 2a). Compounds whose levels significantly changed between IBS patients and healthy controls include many metabolites from food, such as γ-tocotrienol, myo-Inositol 1-phosphate, stearic acid, and actinidine, indicating that some detected changes of metabolite were attributed to the differences in dietary habits (Fig. 2b). Although total serum TBA level increased in IBS patients (Supplementary Table 1), we observed depletions in bile alcohol 27-Norcholestanehexol and bile salt Taurochenodeoxycholate-3-sulfate. This discrepancy may be due to different enzymes involved in the metabolism and synthesis of different types of bile acids [32]. Another control-enriched metabolite, Tetrahydrodeoxycorticosterone (THDOC), is a stress-induced neuroactive and anti-oxidative steroid, which might protect stress-induced responses [33]. Our result showed significant negative correlations between serum levels of THDOC with SDS in IBS (Supplementary Fig. 8a).

Considering the simultaneous elevation of four fatty acyl-CoAs in IBS patients, we hypothesized that metabolites may be clustered for similar chemical and functional properties. Using the unsupervised clustering method based on residual abundance values from linear modelling described in detail by Franzosa et al. [25], 726 differentially abundant serum metabolites were clustered into 78 clusters which tend to covary independently of their relationship with IBS phenotype and age (Supplementary Table 2). Clusters of metabolites can be used to predict properties for unannotated metabolites by transforming knowledge from their annotated partners. The largest cluster enriched in IBS patients contained 70 metabolites (Fig. 2c), which include the three out of the four fatty acyl-CoAs, enhanced the importance of dysregulation of fatty acids in IBS patients. Other metabolites in this cluster included some sterol lipids and structural variants of fatty acid. Moreover, 58 unlabeled metabolites were also contained in this cluster which may also be related to fatty acid metabolism via guilt-by-association logic. The largest cluster enriched in healthy controls contained 123 metabolites, and all of them elevated in healthy controls (Supplementary Fig. 8b). Validated standard metabolites in this cluster included a variety of Triglyceride (TG) metabolites and phosphates. Another interesting cluster enriched in healthy control contained a variety of amines, including Pyridoxamine-5′-Phosphate, Phenylethylamine, and Dimethyltryptamine (Fig. 2d). Pyridoxamine 5′-phosphate is one form of vitamin B6, which is involved in many reactions of amino acid metabolism [34]. Dimethyltryptamine has a similar chemical structure to the neurotransmitter serotonin and acts as an agonist in mammalian brain and blood [35]. Phenylethylamine is a monoamine neurotransmitter, which can stimulate the body to make certain chemicals that play a role in depression and other psychiatric conditions [36]. The co-functions of these organic compounds in IBS still need further research. Most clusters remained largely undefined, allowing the potential correlation analysis for many previously undescribed metabolites with microbial origin.

### Microbiome alterations between IBS patients and healthy controls

Although several previous studies already explored the IBS gut microbiome characteristics in IBS even in large cohort [5, 6], much of the data related to Western populations, limiting their application in a global context [29]. Considering the heterogeneity and complexity of IBS, together with the fact that the prevalence of IBS varies dramatically among geography, it is worth investigating in different countries and cultures. To infer the differences of gut microbiome between IBS patients and healthy controls in Hong Kong Chinese populations, we applied LEfSe [24] to the high-dimensional taxonomic profiles. A total of 33 species were identified as differentially abundant bacteria species, of which 23 were elevated in IBS relative to controls, including *Ruminococcus gnavus, Escherichia coli*, *Bacteroides plebeius*, etc. In contrast, 10 species including *Bacteroides uniformis, Prevotella stercorea*, and *Bacteroides coprocola* were among the species exhibiting the strongest enrichments in healthy controls (Supplementary Fig. 9a and Supplementary Table 4a). Of 33 differentially abundant species in the discovery cohort, 24 trended in the same direction in the validation cohort, suggesting the majority of IBS-associated changes identified in the discovery cohort generalized in their directionality to the validation cohort. Notably, the enrichment of *Bacteroides plebeius* and *Lachnospiraceae bacterium 2_1_58FAA* was also observed in the validation cohort in spite of the small number of differentially abundant species (Supplementary Table 5).

**Fig. 3:**
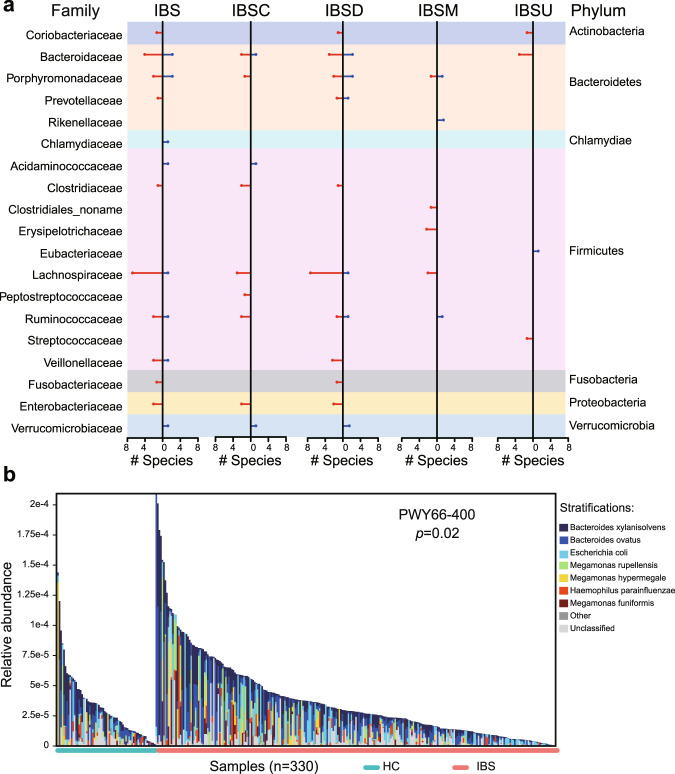
Gut microbiota alterations between IBS patients and healthy controls. By analyzing the gut metagenomic profiles of 264 IBS and 66 HC from the discovery cohort, we identified 33 differentially abundant gut microbiota species, with 23 enriched in IBS and 10 enriched in HC. **a** Differentially abundant gut microbiota species and their belonging taxon at the family level. The *X*-axis labels “# Species” represent the number of differentially abundant species, and the *Y*-axis represents the belonging taxon at the family level. Species that enriched in IBS and IBS subtypes are shown in red, whereas species that enriched in HC are shown in blue. **b** An example of differentially abundant pathways between IBS and HC, annotated by their taxonomic contributors. The *Y*-axis represents the relative abundance of MetaCyc pathway, while the *X*-axis represents ordered individuals from the 330-member discovery cohort.

IBS patients could display different symptoms, for example, diarrhea versus constipation, thus it is important and interesting to figure out the similarities and differences among subtypes in terms of gut microbiome and metabolome. Figure 3a gives an overview of the gut microbiota differentially identified in all IBS clinical subtypes, depicting the numbers of increased and decreased species per family. In total, 16, 29, 9, and 7 nonredundant taxa were associated with IBS-C, IBS-D, IBS-M and IBS-U patients, respectively (Supplementary Fig. 9b−e and Supplementary Table 4b−e). Compared with controls, patients with IBS-C or IBS-D showed substantial overlap in the increase and decrease in the relative abundance of bacterial species in their gut microbiome. There were 10 taxa associated with both IBS-D and IBS-C (Supplementary Table 6). These included an increase in several Gram-negative bacteria, including *Bacteroides faecis, Escherichia coli*, *and Klebsiella pneumoniae*. Furthermore, the *Bacteroides clarus* and *Bacteroides coprocola* showed an opposite changing direction, may be associated with the different symptoms in IBS-C and IBS-D. In addition, we also found some disease-specific associations. The abundance of *Fusobacterium varium*, for example, was only elevated in patients with IBS-D but not in those with IBS-C. An increase in species of the *Clostridium* was observed only in patients with IBS-C, including increases in *Clostridium symbiosum* and *Clostridium bartlettii* [11].

To understand the functional consequences of microbial community changes in IBS, we profiled pathways in all metagenomes using HUMAnN2 [23]. LEfSe [24] was applied on the abundance data, revealing 18 pathways differentially abundant in IBS patients and healthy controls (Supplementary Table 7). Of the differentially abundant pathways, 8 was significantly elevated in IBS patients. The glycolysis VI (mammalian) represented the most significantly enriched functional pathway in IBS, which was dominated by the *Bacteroides* genus (Fig. 3b). The synthesis of stearate, androgen, and L-tyrosine were also enhanced in IBS (Supplementary Fig. 10a, b, and Supplementary Table 7). The most common pathways enriched in healthy controls included biosynthesis of L-lysine (PWY_5097, PWY_2941, PWY_2942, and PWY_724), and the biosynthesis of isoleucine and threonine (Supplementary Fig. 10c−e and Supplementary Table 7). Our functional analysis revealed differential metabolic pathways between IBS patients and healthy controls, and these observations may help to explain the great divergence of serum metabolites between these two groups.

### Associations between fecal/serum metabolites and gut microbiota

**Fig. 4:**
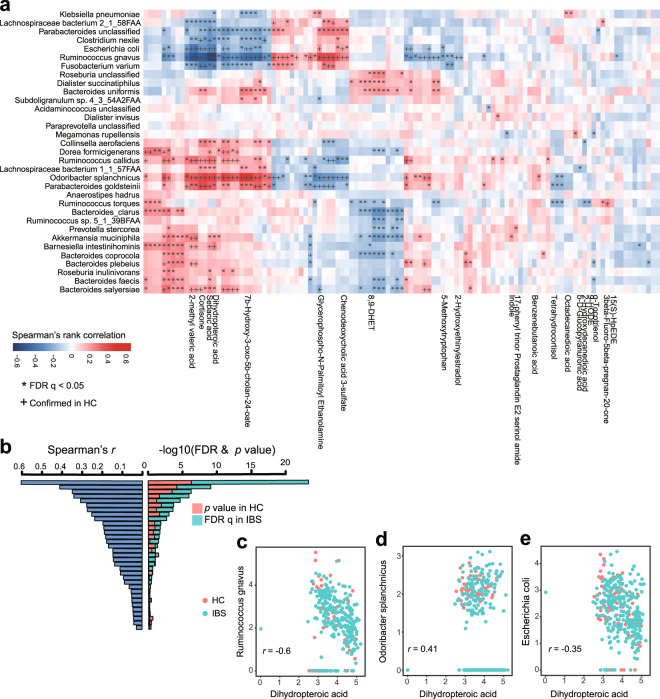
Associations between differentially abundant fecal metabolites and differentially abundant species. By analyzing the fecal metabolomic and gut metabolomic profiles of 264 IBS and 66 HC from the discovery cohort, we identified 113 differentially abundant fecal metabolites (including 21 metabolites that were structurally identified) and 33 differentially abundant species. **a** The heatmap shows the associations between 33 differentially abundant species and 113 differentially abundant fecal metabolites (including 21 structurally known metabolites). **b** The barplot illustrates associations between dihydropteroic acid and 33 differentially abundant species. Each row represents one differentially abundant species. The left panel shows the absolute values of Spearman’s rank correlation coefficients in the discovery cohort, while the right panel shows corresponding *q* values in IBS patients and *p-*value in healthy controls. **c**−**e** Shown are the top 3 differentially abundant species (*Ruminococcus gnavus*, *Odoribacter splanchnicus*, and *Escherichia coli*) and their Spearman’s rank correlation coefficients with dihydropteroic acid.

The multi-omics nature of our data enables the identification of dynamic interaction between differentially abundant microbial and metabolic features in IBS. Positive associations between microbiota and metabolites might suggest metabolites promote the growth of certain species or species produce certain metabolites; while negative associations may suggest competitive or suppressive relationship. Here we analyzed the association between species and fecal/serum metabolites. Interestingly, a total of 522 associations (*q* < 0.05) between 113 differentially abundant fecal metabolites and 33 differentially abundant gut bacteria species were revealed (Fig. 4a); however, the association between differentially abundant serum metabolites and gut bacteria showed no statistical difference. These findings suggested that fecal metabolites, which could directly interact with gut microbiota, in comparison serum metabolites are regulated by more complex and subtle mechanisms. To further confirm potential mechanistic associations that are perturbed in IBS, we specifically focused on the subset of associations that were nominally significant (*p-*value < 0.05) and changes in the same direction in healthy controls. This analysis showed that 30% (155 out of 522) associations could be validated in controls, including 43 associations involving structurally known metabolites (Supplementary Table 8). Of 43 associations, 13 out of 33 differentially abundant species were represented in at least one association. *Ruminococcus gnavus* associates with eight metabolites and followed by species *Odoribacter splanchnicus* and *Escherichia coli* that associate with seven metabolites (Supplementary Table 8). In line with IBD, the over-represented abundance of *Ruminococcus gnavus* is elevated in IBS compared with healthy control, and it is negatively associated with metabolites such as dihydropteroic acid, sebacic acid, 2-methyl valeric acid, and cortisone. Especially, *Ruminococcus gnavus* is strongly associated with dihydropteroic acid (Spearman’s *r* = −0.60), which is an important intermediate product for folic acid. Folic acid is reported to be relatively low in IBS patients. Our result demonstrated that the enriched *Ruminococcus gnavus* was closely related to the low level of folic acid in IBS. In addition, *Odoribacter splanchnicus* and *Escherichia coli* are also observed to associate with dihydropteroic acid (Fig. 4b−e, Spearman’s *r* = 0.41 and −0.35). Our data demonstrated the importance of integrating gut microbiome and metabolome, as it provides an explaining framework for IBS pathogenesis and complication, and identifies microbial and metabolic candidates for further investigation and application.

### Microbial and metabolic signatures associated with IBS depression

**Fig. 5:**
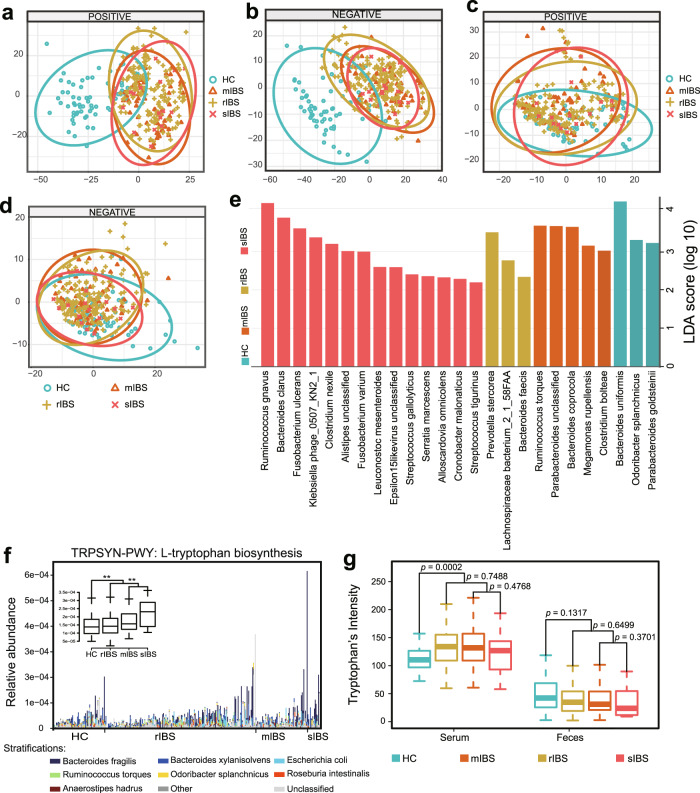
Aberrant metabolomic and metagenomic patterns in IBS patients with and without depression. **a, b** PLS-DA score plots based on the serum metabolomic profiles of 259 IBS patients (including 181 rIBS, 62 mIBS, and 16 sIBS) and 66 healthy controls form the discovery cohort in both positive and negative ionization modes. **c, d** PLS-DA score plots based on fecal metabolomic profiles of 264 IBS patients (including 185 rIBS, 63 mIBS, and 16 sIBS) and 66 healthy controls form the discovery cohort in both positive and negative ion modes. **e** Differentially abundant gut microbiota species among rIBS, mIBS, sIBS, and HC, by using LEfSe to analyze the gut metagenomic profiles of individuals from the discovery cohort. **f** The relative abundance of L-tryptophan biosynthesis pathway in rIBS, mIBS, sIBS, and HC. Here the abundance of TRP production is inferred by analyzing the fecal metagenomic profiles of 264 IBS and 66 HC from the discovery cohort, not yet measured in situ. **g** The signal intensity of tryptophan in serum and feces in rIBS, mIBS, sIBS, and HC, by analyzing the serum and fecal targeted metabolic profiles of individuals from the discovery cohort.

To investigate the potential association between multi-omics signatures and severity of psychological symptoms, especially depression, we classified samples into four groups: healthy controls (HC), regular IBS patients without depression (rIBS: HAMD < 7 and SDS < 53), IBS patients with mild depression (mIBS: 7≤HAMD < 17 and SDS ≥ 53) and IBS patients with moderate or severe depression (sIBS: HAMD ≥ 17 and SDS ≥ 53). By applying PLS-DA, we revealed enormous differences between healthy controls and IBS depression groups (including rIBS, sIBS, and mIBS) (Fig. 5a, b). The serum compositional changes involved 836 and 754 metabolites that altered abundance in mIBS and sIBS, respectively (Supplementary Table 9). Of them, 693 metabolites are shared. The significantly increased compounds include guanine, stearamide, and anandamide. In contrast, we found fewer differences in fecal metabolome between control and depression groups (Fig. 5c, d and Supplementary Table 10). A similar phenomenon was observed in gut microbiota, and only 37 species showed aberrant alterations (Fig. 5e and Supplementary Table 11). However, functional analysis revealed a gradually increasing enrichment of L-tryptophan biosynthesis pathway across control, rIBS, mIBS, and sIBS groups (Fig. 5f and Supplementary Table 12).

To identify metabolic and metagenomic features that distinguish depressed-IBS from regular subjects, we further make a comparison among rIBS, mIBS, and sIBS groups. The multi-omics divergence between rIBS and the depressed group was smaller than that compared with healthy controls. Notably, the sIBS patients showed more divergence than mIBS patients (Supplementary Fig. 11 and Supplementary Tables 13−15). Interestingly, the L-tryptophan biosynthesis pathway was over-represented in sIBS patient compared with rIBS (Supplementary Table 16). We also quantified depression-related molecules using a targeted metabolic profiling. Consistent with the enhanced TRP biosynthesis ability in gut microbiota, the TRP intensity also significantly increased in serum (Fig. 5g). In addition, we also noticed that there are some other elevated compounds in the depression group compared with healthy controls, including histamine, tryptamine, kynurenine (KYN) (Supplementary Fig. 12).

To explore the associations between neuroactive amino acids/neurotransmitters and gut microbiota, we selected eight representative species which have the greatest power to distinguish IBS patients with depression from healthy controls (Supplementary Fig. 13a, b) and examined their relationships with these molecular compounds. A positive association of *Roseburia inulinivorans* and histamine was observed in both fecal and serum data (Supplementary Fig. 13c), indicating a promoting role of *Roseburia inulinivorans* in the production of histamine. In addition, *Roseburia inulinivorans* was associated with melatonin, tryptamine, 3-HAA, KYN, glutamine, and dopamine in serum, although these associations are not replicated in feces. Furthermore, the IBS-enriched species, *Clostridium nexile*, correlate with a broader range of metabolite changes, such as NAS, TRP, and 5-HIAA. Taken together, our data suggested that gut microbiome producing amino acids and amines, such as TRP, serotonin, and histamine, could be involved in the synthesis and degeneration of many neurotransmitters thus affecting the host’s mood and psychological conditions. The integration of metagenome and metabolome data enables us to connect certain bacteria and associated metabolic products, providing new clues to understand IBS depression comorbidity.

### Applications of multi-omics features in IBS detection

Currently, IBS is basically diagnosed based on symptoms, lacking valid biomarkers. We thus explored the performance of using microbiome and/or metabolites features to distinguish IBS patients from healthy controls, and to detect different subtypes. Here we built RF models using the filtered relative abundance of microbiome and metabolites features and 10-fold cross-validation in the discovery and the independent validation cohorts.

**Fig. 6:**
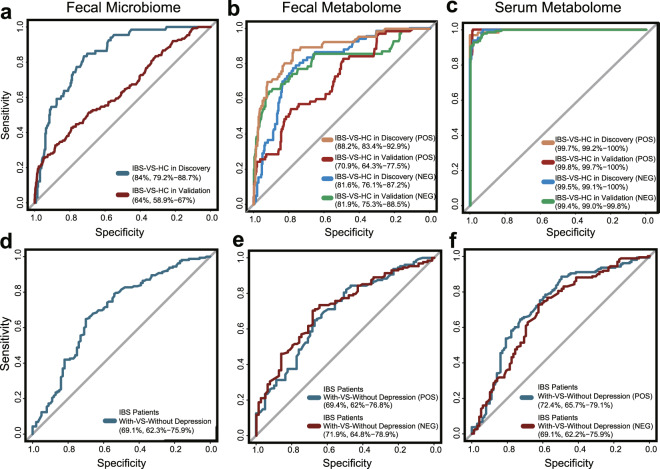
Predicting depression status of IBS patients from multi-omics features. **a** Receiver operating characteristic curve (ROC) of the RF model using gut microbiota profiles to classify IBS patients from healthy controls in discovery and validation cohorts. **b** ROC of the RF model using the fecal metabolomic profiles to classify IBS patients from healthy controls in discovery and validation cohorts. **c** ROC of the RF model using serum metabolome data to classified IBS patients from healthy controls in discovery and validation cohorts. **d**−**f** ROC of the RF model using gut microbiota, fecal metabolomic, and serum metabolomic data, respectively, to train and predict depression status of IBS patients. AUC value is presented in the format of 95% confident interval. For serum and fecal metabolomic data, only the possibility distributions derived from untargeted positive ion mode are presented.

Based on fecal microbiome data, the AUCs for IBS detection were 0.839 [95% confidence interval (CI): 79.2−88.69] in the discovery cohort, and 0.639 (95% CI: 58.93−68.99) in the validation cohort (Fig. 6a). The AUCs for IBS-D detection were 0.855 (95% CI: 80.81−90.14) in the discovery cohort, and 0.746 (95% CI: 70.52−78.6) in the validation cohort (Supplementary Fig. 14a, b). The same procedure was applied to IBS-C, IBS-M, and IBS-U, but only the result of IBS-C was shown due to the limited sample size for IBS-M and IBS-U. Based on fecal metabolome data, the AUCs for IBS detection were 0.882 (95% CI: 83.37−92.93) in the discovery cohort, and 0.709 (95% CI: 64.33−77.46) in validation set (Fig. 6b). A similar trend could be observed in IBS-D detection, with an AUC of 0.880 (95% CI: 82.66−93.29) in the discovery cohort, and 0.671 (95% CI: 60.08−74.15) in the validation cohort (Supplementary Fig. 14c, d). Based on serum metabolome data, IBS patients were almost perfectly detected from healthy controls with an AUC of 0.997 (95% CI: 99.17−100) (Fig. 6c) in the discovery cohort, and 0.998 (95% CI: 99.67−99.95) in the validation cohort. IBS-D could be superiorly predicted from healthy controls as well, achieving an AUC of 0.996 (95% CI: 99.15−100) in the discovery cohort, and 0.997 (95% CI: 99.49−99.92) in the validation cohort (IBS-D = 57, control = 15) (Supplementary Fig. 14e, f). Similar trends could be replicated in IBS-C, IBS-M, and IBS-U despite limited sample numbers. All these supervised learning results were consistent with our unsupervised PCoA analysis using serum metabolites (Supplementary Fig. 5c and  6c), suggesting that serum metabolites represented good predictors to distinguish IBS patients from healthy controls. It was worth noting that the unbalanced sample size among subtypes impaired the efficacy to identify subtype-specific microbiome/metabolome features, as well as their potential to diagnose different subtypes. Therefore, we only found the mediocre performance of metabolome data among inter-subtypes models, such as the IBS-D versus IBS-C (Supplementary Fig. 14e, f).

Since IBS is often accompanied by anxiety and depression, there were investigations attempt to differentiate depression status using microbiota [37]. Here in this study, using fecal microbiota data, the AUC for predicting IBS patients with depression was 0.691 (95% CI: 62.31−75.89) (Fig. 6d). After stratifying IBS patients into rIBS (*n* = 162), mIBS (*n* = 81) and sIBS (*n* = 17) subgroups, the AUC for predicting each subgroup from healthy controls was greater than 0.8 (Supplementary Fig. 15a). Specifically, distinguishing rIBS from mIBS showed a relatively low AUC of 0.639 (95% CI: 56.76−70.96), indicating mild microbiota shift from rIBS to mIBS (Supplementary Fig. 15b). Similar trends were observed using fecal metabolome data (Fig. 6e and Supplementary Fig. 15c, d). Whereas based on serum metabolome data, the AUCs for predicting IBS patients with depression were 0.724 (95% CI: 65.73−79.07) and 0.691 (95% CI: 62.24−75.93) in positive and negative mode, respectively (Fig. 6f). All the subgroups including rIBS (*n* = 162), mIBS (*n* = 81) and sIBS (*n* = 17) could be distinguished from control, achieving the AUCs of 0.990 and even higher (Supplementary Fig. 15e). These results indicated that metabolome data may not be good predictors for IBS depressive status, although it could clearly distinguish IBS patients from healthy controls.

## Discussion

This study reported large-scale integrated analysis of microbiome and fecal/serum metabolome of IBS patients for the first time. Compared with other diseases’ relationship with microbiome, IBS patients showed moderate alterations in both gut microbiome and fecal metabolome compared to that of healthy controls, and there was no clear separation observed between IBS patients and healthy controls. Although we did not observe significant changes in the microbiome of IBS patients, we did notice 23 IBS enriched species and eight IBS enriched functional pathways. The elevated glycolysis VI (mammalian) pathway may suggest abnormalities in energy metabolism involved in IBS; while the elevated synthesis stearate, androgen, and L-tyrosine pathways may increase the sensitivity of tyrosine receptor kinase receptors, associating with adjustment of neuronal transmission strength [38]. The biosynthesis of L-lysine represented a functional pathway that enriched in healthy controls but downregulated in IBS patients, and it was reported to act as a partial serotonin receptor 4 antagonist and inhibit serotonin-mediated intestinal pathologies and anxiety [39]. Moreover, tea drinking was observed to be associated with fecal microbiota and metabolites in IBS, which is consistent with a previous study that tea could have an effect on gut microbiota [40]. However, more detailed relationship between tea drinking and IBS still needs further research. In contrast to fecal microbiome and metabolome, serum metabolites demonstrated clearly separation of IBS patients from healthy controls. We observed the serum metabolite THDOC was enriched in healthy controls and depleted in IBS, and identified negative correlation between serum level of THDOC with SDS in IBS. This phenomenon is similar to a previous study that reported reduced serum concentration of THDOC in women during menstruation epilepsy with depression [41], suggesting the metabolite may be involved in the interaction between depression and IBS. In addition, our data revealed that nearly 50% of the serum metabolites exhibit significant changes even when adjusting for the linear influence of dietary habits, and these data might provide important implications for IBS diagnosis. It is worth noting that we tested the influence of dietary habits using a linear model. We cannot rule out non-linear influences as the interactions between diet and serum metabolome are complicated and not well understood yet.

According to the results, there are certain associations between metabolic dysregulation and IBS pathogenesis. For example, guanine represented the most abundant metabolite in IBS patients. It was proposed that guanine is involved in a specific guanine-based purinergic system which is able to affect the development and structure of neural cells in central nervous system and correlated with memory and anxiety [42]. The largest IBS-enriched serum metabolite cluster included three fatty acyl-CoAs (tetradecanoyl-CoA, myristoleoyl-CoA, and lauroyl-CoA), suggesting the dysregulation of fatty acid profile may involve in IBS and depression [16, 43]. Chua et al. showed omega-3 polyunsaturated fatty acids deficiency in IBS which was ascribed to substantial effects on the nervous systems. Clarke et al. [44] suggested omega-3 supplementation may be a putative treatment for IBS. Previously, a population-based cross-sectional study in IBS had found it was positively correlated to metabolic syndrome [45], reinforcing the metabolic dysregulation in IBS. In addition, we found gut microbiota species associated with dihydropteroic acid, which is an intermediate product for folic acid. As 11 out of 33 differentially abundant species are associated with dihydropteroic acid, there is a possibility that IBS-featuring microbiome could affect disease by modulating the metabolism of folic acid. However, it is only an inference based on data analyzing, and not yet measured in cohort study. Further studies are needed to confirm the potential relationship and biological mechanism between these species, folic acid, and the pathogenesis of IBS.

Our results suggested a correlation between TRP/serotonin metabolism and IBS depression comorbidity. The irregularity of TRP in serum was noticed in IBS-D [46], but its role in IBS-relevant depression and associated gut microbiome alterations remains elusive. We identified certain gut bacteria strains such as *Clostridium nexile* and *Roseburia inulinivorans* enriched in IBS patients with depression, and associated with neuroactive TRP metabolites alterations in serum. *Clostridium nexile* could produce neurotransmitter tryptamine, and some other *Clostridium* species, such as *C. diffcile*, have been reported to increase in patients with major depressive disorder [47, 48]. Functional analysis indicated the abundance of L-tryptophan pathway was elevated along with the severity of depression. We also noticed that there are some other elevated compounds in the depression group compared with healthy controls, including kynurenine (KYN), tryptamine, and histamine. Although more than 90% of the body’s serotonin is produced in the gut by enterochromaffin cells under the influence of the gut microbiota, peripheral serotonin does not cross the blood-brain barrier under physiological conditions. The neurotransmitter serotonin is produced locally in the brain through the enzyme TRP hydroxylase 2 (TpH2). However, it is worth noting that certain gut bacteria strains can directly regulate TRP utilization consequently changing its availability to the host, impacting serotonin levels not only in the periphery but also in the brain. It is widely accepted that there are three major TRP metabolism pathways in the gut, leading to serotonin, KYN, and indole derivatives, respectively. In the case of KYN-producing pathway overactivation, TRP is massively diverted to KYN production, causing a deficiency in brain TRP and in neurotransmitter serotonin production, subsequently leading to depression. Extensive KYN across the blood-brain barrier is also considered critical to central nervous system disorders. Such cases have been reported in several diseases. For example, in a study regarding depression in obesity [49], the authors reported a condition characterized by chronic inflammation and KYN activation that contributed to depression. In a study of autistic children [50], it demonstrated abnormal TRP characterized by preferential transformation from TRP to kynurenic acid. Our finding that KYN was elevated in the depression group compared with healthy controls further supports the overactivation of KYN-producing pathway, suggesting that gut microbiota may indirectly affect central serotoninergic pathways by modulating TRP availability.

We acknowledged some limitations here in this study. Firstly, we did not set a strategy of matching patient number among subtypes of IBS when doing subject recruitment. Analytical results displayed a certain bias due to the vast predominance of IBS-D in the discovery cohort, thus impaired the possibility to identify subtype-specific microbiome and metabolome characteristics, especially for IBS-M and IBS-U. A more balanced sample size among subtypes is needed for subtype-specific feature identification. Secondly, most differentially abundant species identified in the discovery cohort only trended in the same direction but did not achieve significance in the validation cohort. Considering that gut microbiota is affected by multiple factors, and that both the discovery and validation cohort represented combinations of different IBS subtypes and included high heterogeneity, the different fecal traits during diarrhea and constipation would probably affect the robustness of IBS related microbiota features and impair their application in disease classification. In comparison, serum metabolome showed more stable IBS specific characteristics in both the discovery and validation cohort, thus have great power to discriminate IBS patients from healthy controls. Larger cohort would help to better characterize the complicated gut microbiome alterations. Thirdly, our results suggested a correlation between TRP/serotonin metabolism and IBS depression comorbidity. We proposed a hypothesis that gut microbiota of IBS patients with depression might favor TRP/serotonin metabolism along the KYN-producing pathway thus affecting the TRP availability and serotonin levels in the brain. However, this is only an inference based on literature. Considering that the TRP/serotonin mediated gut-brain cross-talk is complex and remains poorly understood, extensive investigations based on in vitro and in vivo models are needed to uncover the exact mechanism.

Finally, IBS is a collection of gastrointestinal syndromes with no clear pathogenesis observed in certain organs. The partially understood pathophysiology, and consequently the absence of universally accepted biomarkers, severely limited efficient diagnosis and therapeutic approaches. Our multi-omics study revealed IBS specific serum/fecal metabolome alterations and their relationship with gut microbiome, highlighted the massive alterations of serum metabolites which empower to recognize IBS patients, suggested potential roles of metabolic dysregulation of fatty acids and folic acid in IBS pathogenesis, and offered new clues to understand IBS depression comorbidity. Our study provided a valuable resource for future studies to understand host-gut microbiota relationships in IBS, and would facilitate potential clinical applications of IBS featured microbiota and (or) metabolites.

## Supplementary information

SupplementaryNotes.docx

Supplementary Figure 1

Supplementary Figure 2

Supplementary Figure 3

Supplementary Figure 4

Supplementary Figure 5

Supplementary Figure 6

Supplementary Figure 7

Supplementary Figure 8

Supplementary Figure 9

Supplementary Figure 10

Supplementary Figure 11

Supplementary Figure 12

Supplementary Figure 13

Supplementary Figure 14

Supplementary Figure 15

Supplementary Tables 1

Supplementary Tables 2

Supplementary Tables 3

Supplementary Tables 4

Supplementary Tables 5

Supplementary Tables 6

Supplementary Tables 7

Supplementary Tables 8

Supplementary Tables 9

Supplementary Tables 10

Supplementary Tables 11

Supplementary Tables 12

Supplementary Tables 13

Supplementary Tables 14

Supplementary Tables 15

Supplementary Tables 16

## Data Availability

Fecal metagenomic sequencing reads can be downloaded from CNGB Nucleotide Sequence Archive under accession number CNP0000334. Serum and fecal metabolome datasets are available at the MetaboLights database under accession number MTBLS2774. Other supporting data can be found in supplementary materials.
