# Supplementary material for: Altered metabolome and microbiome features provide clues in understanding irritable bowel syndrome and depression comorbidity
Source: ISME J. 2021 Nov 8;16(4):983–96. doi: 10.1038/s41396-021-01123-5 (PMC8940891; doi:10.1038/s41396-021-01123-5)
Supplement: Supplementary file 1 — SupplementaryNotes.docx [file 41396_2021_1123_MOESM1_ESM.docx]

**Supplementary Notes**

**Supplementary Figure 1: Overall design of the study.** There were 330 and 101 members recruited for the discovery and validation cohorts, respectively, with fecal and serum samples collected. Specifically, we collected 330 fecal samples (including 24 IBS-C, 214 IBS-D, 19 IBS-M, 7 IBS-U and 66 healthy controls) and 325 matched serum samples (including 24 IBS-C, 209 IBS-D, 19 IBS-M, 7 IBS-U and 66 healthy controls) from the discovery cohort, and collected 101 fecal samples (including 7 IBS-C, 57 IBS-D, 13 IBS-M, 9 IBS-U and 15 healthy controls) and 98 matched serum samples (including 7 IBS-C, 55 IBS-D, 13 IBS-M, 9 IBS-U and 14 healthy controls) from the validation cohort. Fecal metagenomic, fecal metabolomic, and serum metabolomic data were then profiled and analyzed.

**Supplementary Figure 2:** **The inter-correlation between intrinsic and questionnaire factors.** The top panel (a) shows the inter-correlation (Spearman correlation) between 24 intrinsic factors. The bottom panel (b) shows the inter-correlation (Spearman correlation) between 45 questionnaire factors. Here the correlation analysis was performed based on factors that collected from the 330-member discovery cohort.

**Supplementary Figure 3: The comparison of alpha and beta diversity.** The Shannon index and Bray-Curtis distance were calculated based on discovery cohort individuals using 325 serum metabolomic (a), 330 fecal metabolomic (b) and 330 gut metagenomic (c) profiles, respectively. Black bars indicate statistical significance (FDR < 0.1). POS and NEG represent the positive and negative ionization modes in mass spectrometry, respectively.

**Supplementary Figure 4: The correlation of factors and serum/fecal metabolomic profiles.** **(a)** The correlation of factors and fecal metabolomic profiles (positive mode) of 264 IBS and 66 HC from the discovery cohort. **(b)** The correlation of factors and serum metabolomic profiles (positive mode) of 259 IBS and 66 HC from the discovery cohort. **(c)** The barplot shows the correlations between factor Tea Frequency and known fecal metabolites with FDR < 0.1. The Y axis represents associated fecal metabolites, while the X axis represents the Spearman correlation coefficient *r*.

**Supplementary Figure 5: Differentially abundant serum metabolites in negative mode between IBS patients and healthy controls.** In negative mode, we totally captured 890 serum metabolites by analyzing the serum metabolomic profiles of 259 IBS and 66 HC from the discovery cohort, and identified 362 differentially abundant serum metabolites between IBS and HC. **(a)** Heatmap and hierarchical clustering of 362 differentially abundant serum metabolites in negative mode. **(b)** PLS-DA score plots constructed with 890 serum metabolites on 259 IBS (orange points) and 66 HC (blue points) from the discovery cohort. **(c)** PCoA of 325 discovery cohort individuals with serum sample collected, basing on the 362 differentially abundant serum metabolites in negative mode.

**Supplementary Figure 6:** **Differentially abundant serum metabolites in positive mode between IBS patients and healthy controls.** In positive mode, we totally captured 879 serum metabolites by analyzing the serum metabolomic profiles of 259 IBS and 66 HC from the discovery cohort, and identified 364 differentially abundant serum metabolites between IBS and HC. **(a)** Heatmap and hierarchical clustering of 364 differentially abundant serum metabolites in positive mode. **(b)** PLS-DA score plots constructed with 879 serum metabolites on 259 IBS (orange points) and 66 HC (blue points) from the discovery cohort. **(c)** PCoA of 325 discovery cohort individuals with serum sample collected, basing on the 364 differentially abundant serum metabolites in positive mode.

**Supplementary Figure 7:** **Differentially abundant fecal metabolites between IBS patients and healthy controls.** We totally captured 932 and 856 fecal metabolites in negative and positive modes, respectively, by analyzing the fecal metabolomic profiles of 264 IBS and 66 HC from the discovery cohort. There were 86 and 27 differentially abundant fecal metabolites identified using PLS-DA methods in negative and positive modes, respectively. **(a)** Heatmap and hierarchical clustering of 86 differentially abundant fecal metabolites in negative mode, among which only 7 metabolites showed significantly differences (marked in color). **(b)** Heatmap and hierarchical clustering of 27 differentially abundant fecal metabolites in positive mode, among which only 1 metabolite showed significantly differences (marked in color). **(c)** PLS-DA score plots constructed with 932 fecal metabolites identified in negative mode on 264 IBS (orange) and 66 HC (blue). **(d)** PLS-DA score plots constructed with 856 fecal metabolites identified in positive mode on 264 IBS samples (orange) and 66 control samples (blue).

**Supplementary Figure 8:** **Examples of differentially abundant metabolites and clusters.** **(a)** The negative correlations between serum level of THDOC and SAS/SDS. The given *r* values indicate Spearman’s rank correlation. **(b)** The largest cluster enriched in healthy controls contained 123 metabolites, all of which are significantly elevated in healthy controls.

**Supplementary Figure 9: Differentially abundant gut microbiota species between IBS patients and healthy controls.** Here the differentially abundant species analysis was performed based on the metagenomic profiles of 330 fecal samples that collected from the discovery cohort, including 264 IBS patients (24 IBS-C, 214 IBS-D, 19 IBS-M and 7 IBS-U) and 66 healthy controls. **(a)** Differentially abundant species identified between 264 IBS patients and 66 HC. In total we identified 33 differentially abundant gut microbiota species between IBS and HC, with 23 enriched in IBS and 10 enriched in HC. **(b)** Differentially abundant species identified between 24 IBS-C patients and 66 HC. **(c)** Differentially abundant species identified between 214 IBS-D patients and 66 HC. **(d)** Differentially abundant species identified between 19 IBS-M patients and 66 HC. **(e)** Differentially abundant species identified between 7 IBS-U patients and 66 HC.

**Supplementary Figure 10: Examples of differentially abundant pathways between IBS patients and healthy controls. (**a, b) represent examples of differentially abundant MetaCyc pathways that enriched in IBS; (c-g) represent examples of differentially abundant MetaCyc pathways that depleted in IBS. The Y axis represents the relative abundance of MetaCyc pathways, while the X axis represents ordered individuals from the 330-member discovery cohort, including 264 IBS and 66 HC. Pathways were further annotated by their taxonomic contributors.

**Supplementary Figure 11: The aberrant metabolomic and metagenomic patterns in IBS patients with and without depression.** **(a)** The aberrant serum metabolomic patterns in IBS patients with and without depression. The analysis was performed based on the serum metabolomic profiles of 259 IBS serum samples that collected from the discovery cohort, including 181 IBS patients without depression (rIBS), 62 IBS patients with mild depression (mIBS) and 16 IBS patients with moderate or severe depression (sIBS). **(b)** The aberrant fecal metabolomic patterns in IBS patients with and without depression. The analysis was performed based on the fecal metabolomic profiles of 264 IBS fecal samples that collected from the discovery cohort, including 185 rIBS, 63 mIBS and 16 sIBS. **(c)** The aberrant fecal metagenomic patterns in IBS patients with and without depression. The LEfSe analysis was performed based on the fecal metagenomic profiles of 264 IBS fecal samples that collected from the discovery cohort, including 185 rIBS, 63 mIBS and 16 sIBS.

**Supplementary Figure 12:** **The signal intensity of these neuroactive molecules in serum and fecal molecule neurotransmitters.** We quantified 17 depression-related neurotransmitters, including TRP, TRPT, NAS, 5-HIAA, melatonin, KYN, KYA, serotonin, 3-IAA, 3-HAA, TYR, SUCC, dopamine, GLU, GLN, histamine, and GABA, using targeted metabolic profiling methods. Shown are the signal intensities of 17 neuroactive molecules and relevant ratios in serum samples **(a)** and in fecal samples **(b)** from discovery cohort, respectively.

**Supplementary Figure 13:** **The selection of representative species to distinguish IBS patients with depression (sIBS) from healthy controls. (a)** Distribution of 5 trials of 10-fold cross-validation (CV) error in RF classification with the increasing of variables. **(b)** Illustration of 8 representative species that have the greatest power to distinguish IBS patients with depression (sIBS) from healthy controls. **(c)** Correlation of 8 representative species with 17 semi-quantified neuroactive metabolites (including TRP, TRPT, NAS, 5-HIAA, melatonin, KYN, KYA, serotonin, 3-IAA, 3-HAA, TYR, SUCC, dopamine, GLU, GLN, histamine, and GABA) in serum and fecal samples.

**Supplementary Figure 14: Classification of IBS subtypes.** **(a)** Using microbiome data to distinguish IBS-D, IBS-C and HC in discovery and validation cohorts. **(b)** Scatter plot of possibility distribution corresponding to ROC curve “IBS-D_VS_IBS-C in Discovery” in (a). **(c)** Using fecal metabolome data under negative (NEG) and positive (POS) modes to distinguish IBS-D from healthy controls in discovery and validation cohorts. (d) Scatter plot of possibility distribution corresponding to ROC curve “IBS D_VS_HC in Discovery (POS)” in (c). **(e, f)** Using untargeted serum metabolome data under positive ion mode to predicting IBS subtypes in discovery and validation cohorts.

**Supplementary Figure 15:** **Classification of IBS patients with or without depression from healthy controls.** **(a)** Using metagenomic profiles to predict depression status (rIBS, Mibs and sIBS) from healthy controls. **(b)** Using metagenomic profiles to conduct classification between depression statuses (rIBS, mIBS and sIBS). **(c)** Using untargeted fecal metabolomic profiles in positive and negative modes to predict depression status (rIBS, Mibs and sIBS) from healthy controls. **(d)** Using untargeted fecal metabolomic profiles in positive and negative modes to conduct classification between depression statuses. **(e)** Using untargeted serum metabolomic profiles in positive and negative modes to predict depression status (rIBS, Mibs and sIBS) from healthy controls. **(f)** Using untargeted metabolomic profiles in positive and negative modes to conduct classification between depression statuses (rIBS, mIBS and sIBS). All the AUC values are presented in the format of 95% Confident Interval. Positive indicates untargeted positive ion mode, and negative indicates untargeted negative ion mode.

**Supplementary Table 1: Baseline characteristics between IBS patients and healthy controls.** Here the baseline characteristics were counted based on the information collected from the 330-member discovery cohort, which included 264 IBS patients (24 IBS-C, 214 IBS-D, 19 IBS-M and 7 IBS-U) and 66 healthy controls.

**Supplementary Table 2: Differentially abundant serum metabolites between IBS patients and healthy controls.** Here the differentially abundant metabolites analysis was performed based on the metabolomic profiles of 325 serum samples that collected from the discovery cohort, which included 259 IBS patients (including 24 IBS-C, 209 IBS-D, 19 IBS-M and 7 IBS-U) and 66 healthy controls. We identified 726 differentially abundant serum metabolites, among which 101 out of the 726 metabolites were structurally identified.

**Supplementary Table 3: Validation of differentially abundant serum metabolites in the validation cohort.** Here we validated 726 differentially abundant serum metabolites based on the metabolomic profiles of 98 serum samples from the validation cohort, including 84 IBS patients (7 IBS-C, 55 IBS-D, 13 IBS-M and 9 IBS-U) and 14 healthy controls.

**Supplementary Table 4: Differentially abundant species between IBS patients and healthy controls.** Here the differentially abundant species analysis was performed based on the metagenomic profiles of 330 fecal samples that collected from the discovery cohort, including 264 IBS patients (24 IBS-C, 214 IBS-D, 19 IBS-M and 7 IBS-U) and 66 healthy controls. In total we identified 33 differentially abundant gut microbiota species between IBS and HC, with 23 enriched in IBS and 10 enriched in HC.

**(a)** Differentially abundant species identified between 264 IBS and 66 HC.

**(b)** Differentially abundant species identified between 24 IBS-C and 66 HC.

**(c)** Differentially abundant species identified between 214 IBS-D and 66 HC.

**(d)** Differentially abundant species identified between 19 IBS-M and 66 HC.

**(e)** Differentially abundant species identified between 7 IBS-U and 66.

**Supplementary Table 5: Differentially abundant species between IBS patients and healthy controls in validation cohort.** Here the differentially abundant species analysis was performed based on the metagenomic profiles of 101 fecal samples that collected from the validation cohort, including 86 fecal samples from IBS patients (including 7 IBS-C, 57 IBS-D, 13 IBS-M and 9 IBS-U) and 15 fecal samples from healthy controls.

**Supplementary Table 6: Common differentially abundant species associated with both IBS-C and IBS-D.** As mentioned, we identified 16 differentially abundant species between 24 IBS-C and 66 HC from the discovery cohort (Supplementary Table 4b), and 29 differentially abundant species between 214 IBS-C and 66 HC from the discovery cohort (Supplementary Table 4c). Shown are the 10 common differentially abundant species in both IBS-C versus HC and IBS-D versus HC.

**Supplementary Table 7: Differentially abundant pathways between IBS patients and healthy controls.** Here the differentially abundant pathway analysis was performed based on the metagenomic profiles of 330 fecal samples that collected from the discovery cohort, among which 264 fecal samples were from IBS patients (including 24 IBS-C, 214 IBS-D, 19 IBS-M and 7 IBS-U) and 66 fecal samples were from healthy controls. In total we identified 18 differentially abundant pathways between IBS and HC, with 8 enriched in IBS and 10 enriched in HC.

**Supplementary Table 8: Identification of associations between differentially abundant fecal metabolites and species.** As mentioned, by analyzing the fecal metabolomic and gut metabolomic profiles from the discovery cohort, we identified 113 differentially abundant fecal metabolites (including 21 metabolites that structurally identified) and 33 differentially abundant species (Supplementary table 4a). Here we firstly identified 522 associations (*q* < 0.05) between those 113 fecal metabolites and 33 species, subsequently identified 30% (155 out of 522) associations that could be validated in controls, and finally found 43 out of 155 associations involving structurally known metabolites. Shown are the 43 associations between known differentially abundant fecal metabolites and species that can be validated.

**Supplementary Table 9: Differentially abundant serum metabolites between IBS patients with depression and healthy controls.** Here the differentially abundant metabolites analysis was performed based on the metabolomic profiles of 325 serum samples that collected from the discovery cohort, including 181 IBS patients without depression (rIBS), 62 IBS patients with mild depression (mIBS), 16 IBS patients with moderate or severe depression (sIBS) and 66 healthy controls (HC).

**(a)** Differentially abundant serum metabolites between mIBS and HC.

**(b)** Differentially abundant serum metabolites between sIBS and HC.

**Supplementary Table 10: Differentially abundant fecal metabolites between IBS patients with depression and healthy controls.** Here the differentially abundant metabolites analysis was performed based on the metabolomic profiles of 330 fecal samples that collected from the discovery cohort, including 185 IBS patients without depression (rIBS), 63 IBS patients with mild depression (mIBS), 16 IBS patients with moderate or severe depression (sIBS), and 66 healthy controls (HC).

**Supplementary Table 11: Differentially abundant species between IBS patients with depression and healthy controls.** Here the differentially abundant species analysis was performed based on the metagenomic profiles of 330 fecal samples that collected from the discovery cohort, including 185 IBS patients without depression (rIBS), 63 IBS patients with mild depression (mIBS), 16 IBS patients with moderate or severe depression (sIBS), and 66 healthy controls (HC). In total we identified 36 differentially abundant species, with 11 enriched in mIBS, 19 enriched in sIBS and 6 enriched in HC.

**Supplementary Table 12: Differentially abundant pathways between IBS patients with depression and healthy controls.** Here the differentially abundant pathways analysis was performed based on the metagenomic profiles of 330 fecal samples that collected from the discovery cohort, including 185 IBS patients without depression (rIBS), 63 IBS patients with mild depression (mIBS), 16 IBS patients with moderate or severe depression (sIBS), and 66 healthy controls (HC). In total we identified 23 differentially abundant pathways, with 6 enriched in mIBS, 2 enriched in sIBS and 15 enriched in HC.

**Supplementary Table 13:** **Differentially abundant serum metabolites between IBS patients with and without depression.** Here the differentially abundant metabolites analysis was performed based on the metabolomic profiles of 259 IBS serum samples that collected from the discovery cohort, including 181 IBS patients without depression (rIBS), 62 IBS patients with mild depression (mIBS) and 16 IBS patients with moderate or severe depression (sIBS).

**Supplementary Table 14: Differentially abundant fecal metabolites between IBS patients with and without depression.** Here the differentially abundant metabolites analysis was performed based on the metabolomic profiles of 264 fecal samples that collected from the discovery cohort, including 185 IBS patients without depression (rIBS), 63 IBS patients with mild depression (mIBS) and 16 IBS patients with moderate or severe depression (sIBS).

**Supplementary Table 15: Differentially abundant species between IBS patients with and without depression.** Here the differentially abundant species analysis was performed based on the metagenomic profiles of 264 IBS fecal samples that collected from the discovery cohort, including 185 IBS patients without depression (rIBS), 63 IBS patients with mild depression (mIBS) and 16 IBS patients with moderate or severe depression (sIBS).

**Supplementary Table 16: Differentially abundant pathways between IBS patients with and without depression.** Here the differentially abundant pathways analysis was performed based on the metagenomic profiles of 264 IBS fecal samples that collected from the discovery cohort, including 185 IBS patients without depression (rIBS), 63 IBS patients with mild depression (mIBS) and 16 IBS patients with moderate or severe depression (sIBS).

**(a)** Differentially abundant pathways among rIBS, mIBS, and sIBS.

**(b)** Differentially abundant pathways between mIBS and sIBS.

**(c)** Differentially abundant pathways between sIBS and rIBS.
