# Supplementary material for: Altered metabolome and microbiome features provide clues in understanding irritable bowel syndrome and depression comorbidity
Source: ISME J. 2021 Nov 8;16(4):983–96. doi: 10.1038/s41396-021-01123-5 (PMC8940891; doi:10.1038/s41396-021-01123-5)
Supplement: Supplementary file 2 — Supplementary Figure 1 [file 41396_2021_1123_MOESM2_ESM.pdf]

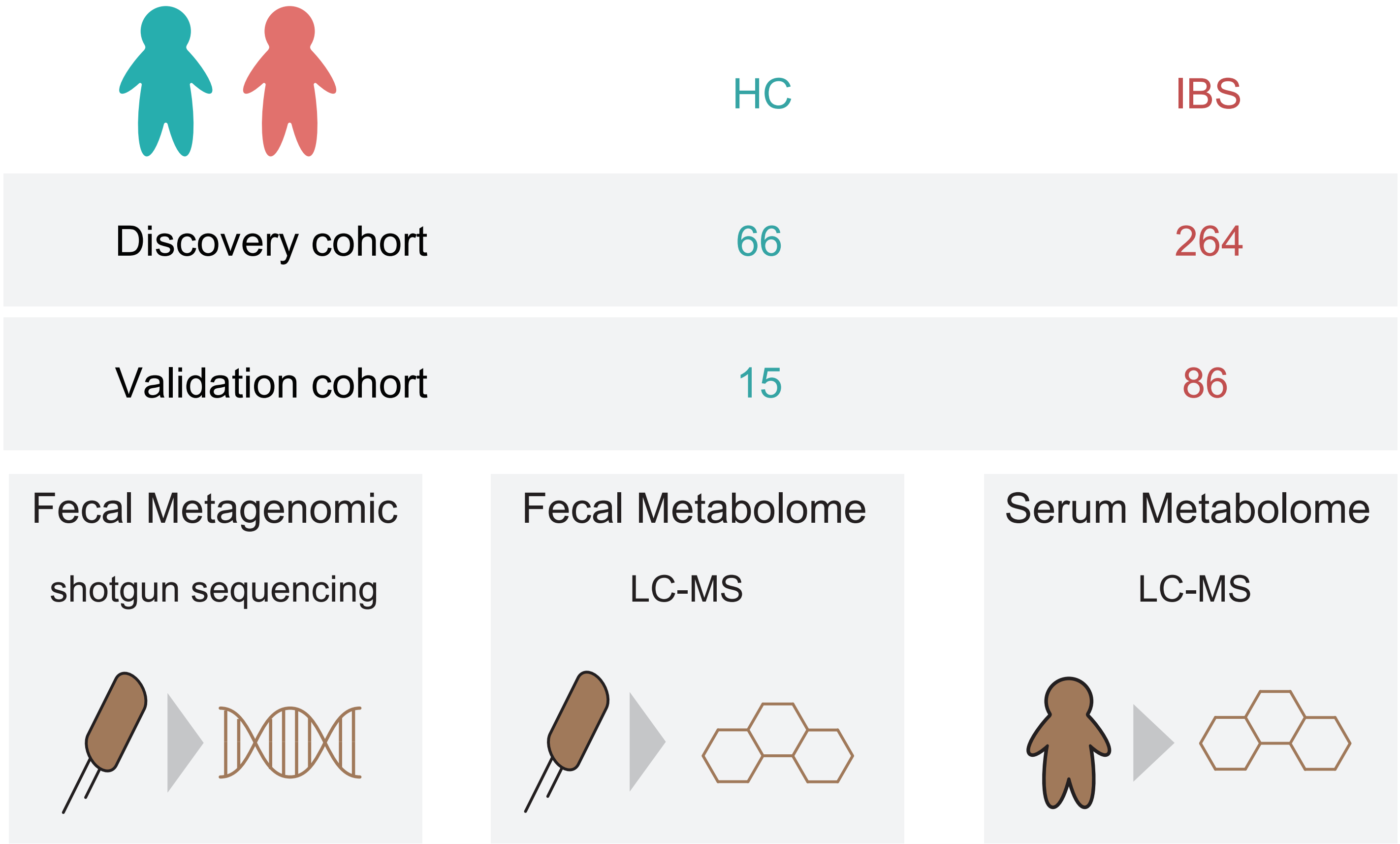
HC
IBS
Discovery cohort
66
264
Validation cohort
15
86
Fecal Metagenomic
shotgun sequencing
Fecal Metabolome
LC-MS
Serum Metabolome
LC-MS
