# Supplementary figures and images for: Altered metabolome and microbiome features provide clues in understanding irritable bowel syndrome and depression comorbidity
Source: ISME J. 2021 Nov 8;16(4):983–96. doi: 10.1038/s41396-021-01123-5 (PMC8940891; doi:10.1038/s41396-021-01123-5)

a

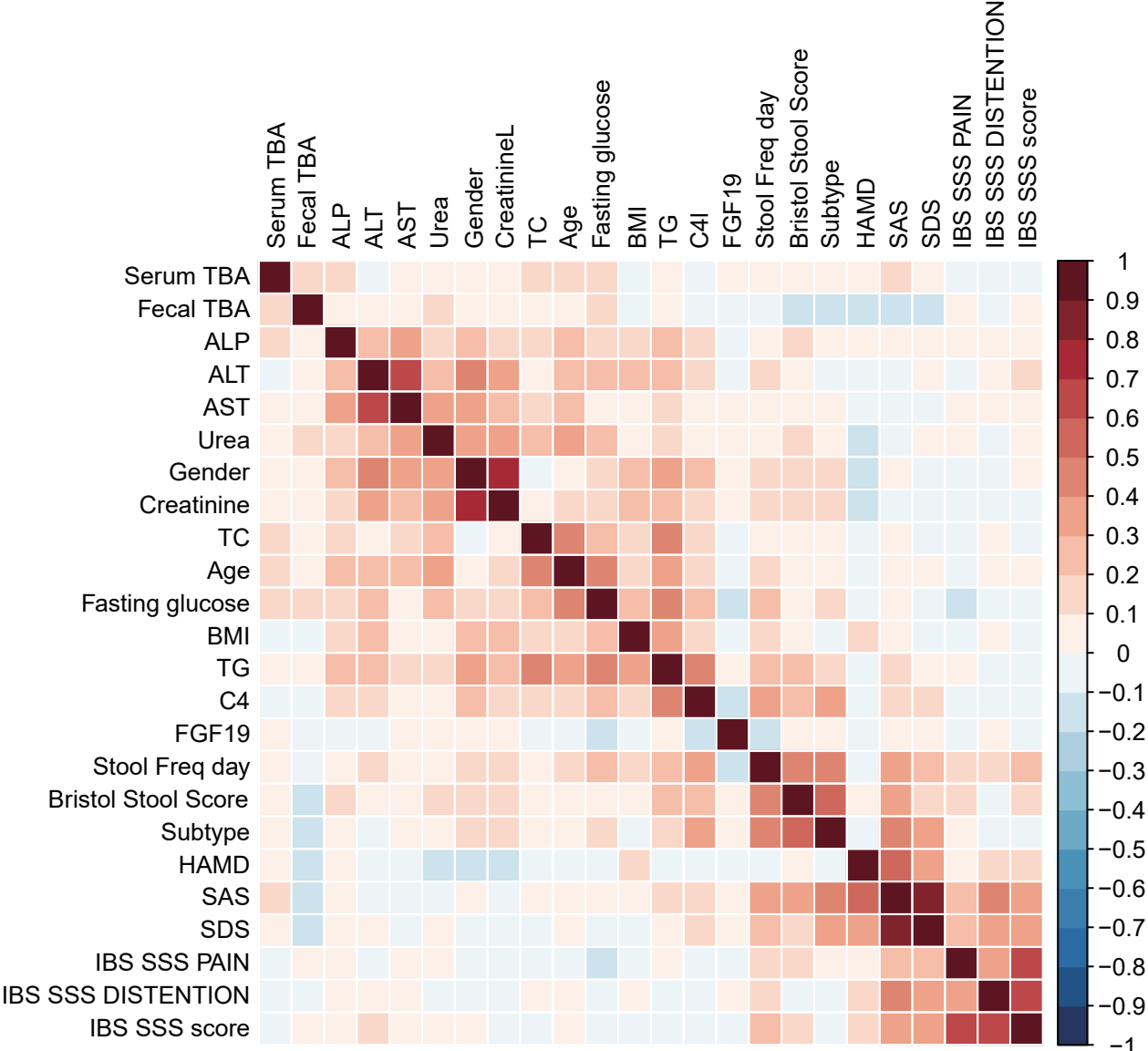

b

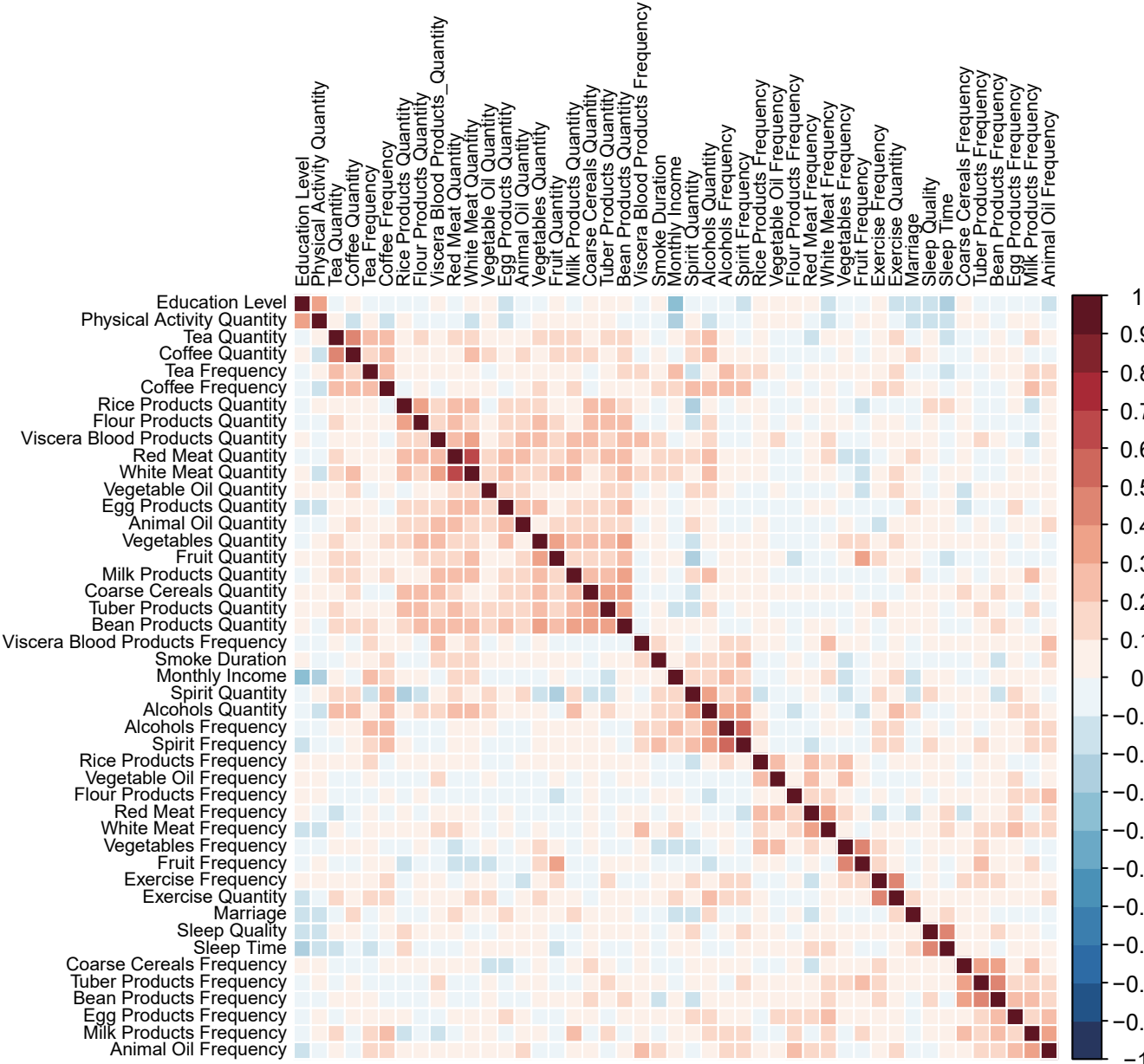

Supplement: Supplementary file 3 — Supplementary Figure 2 [file 41396_2021_1123_MOESM3_ESM.pdf]

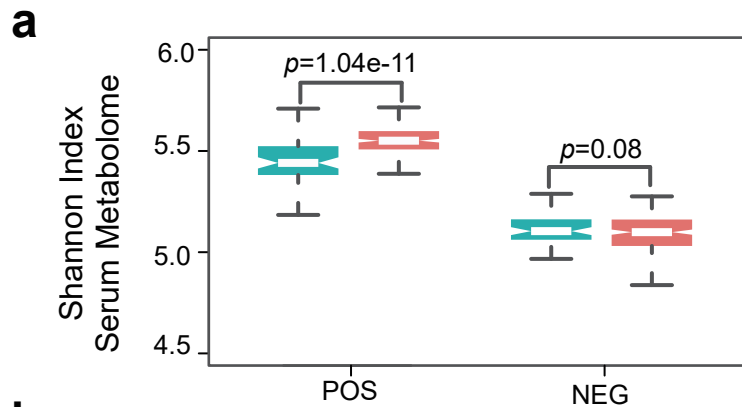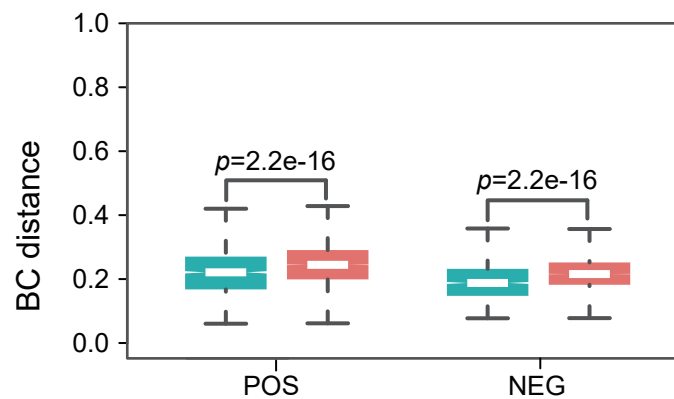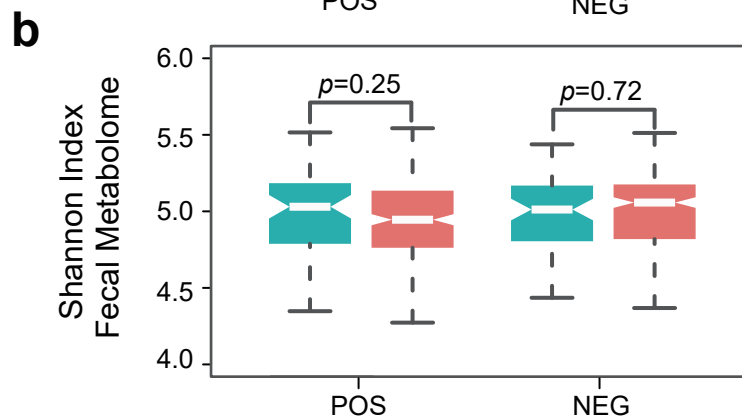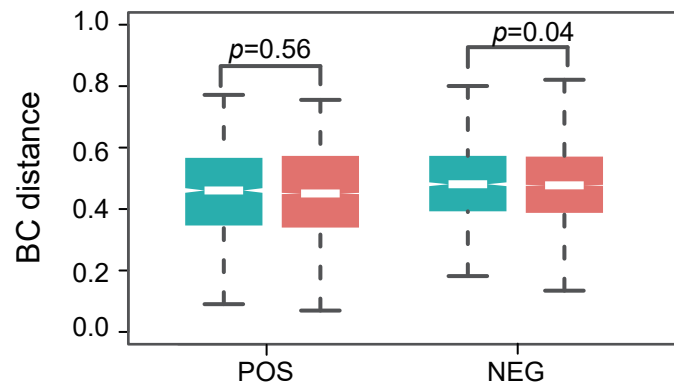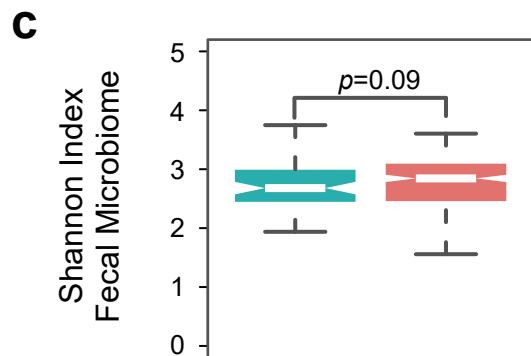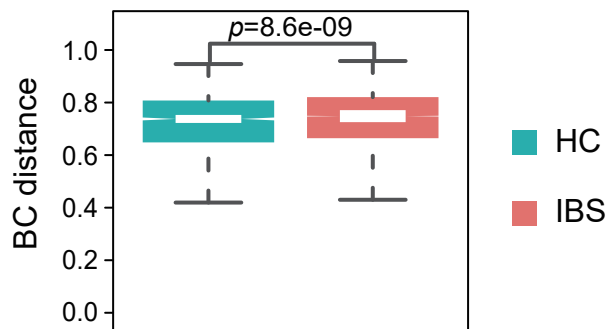

Supplement: Supplementary file 4 — Supplementary Figure 3 [file 41396_2021_1123_MOESM4_ESM.pdf]

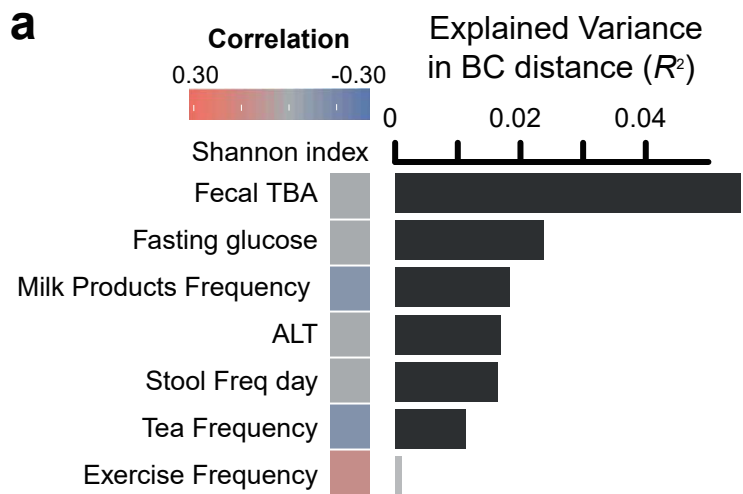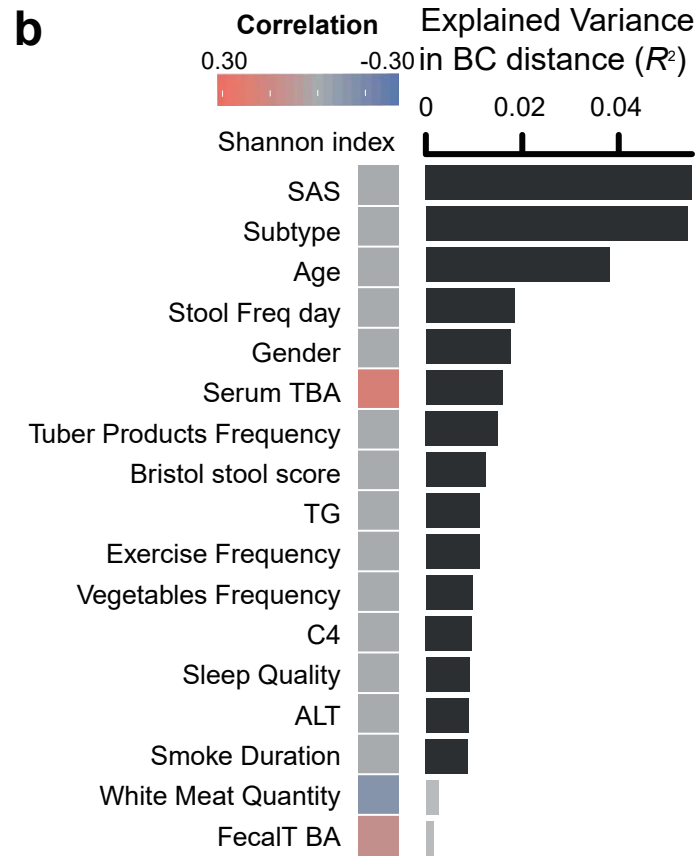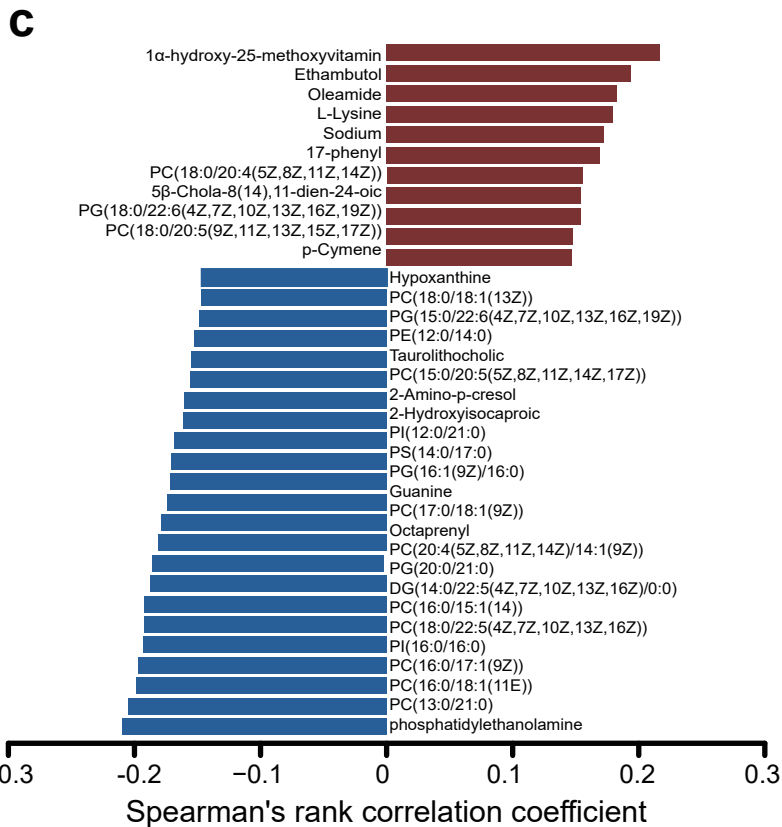

Supplement: Supplementary file 5 — Supplementary Figure 4 [file 41396_2021_1123_MOESM5_ESM.pdf]

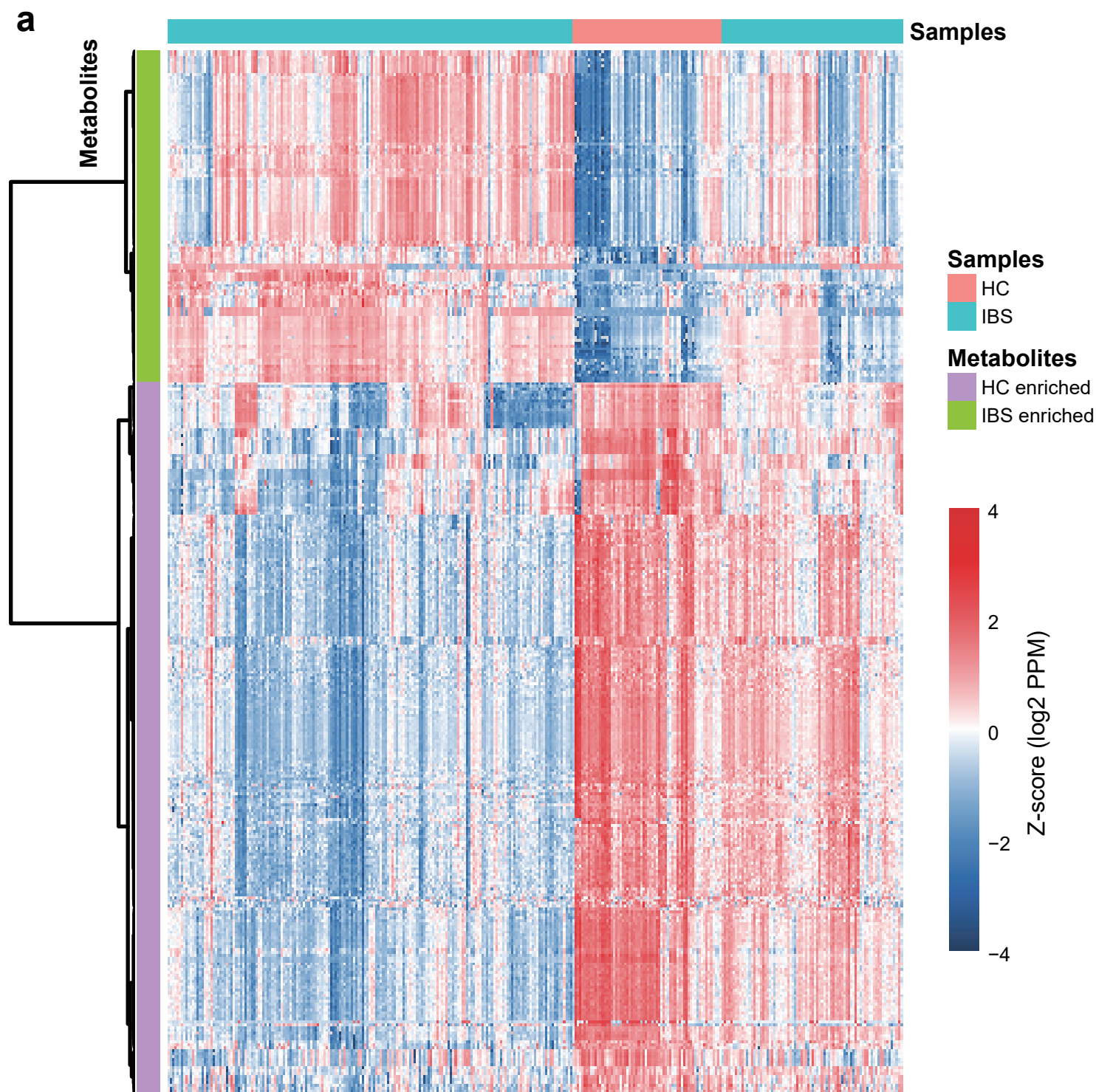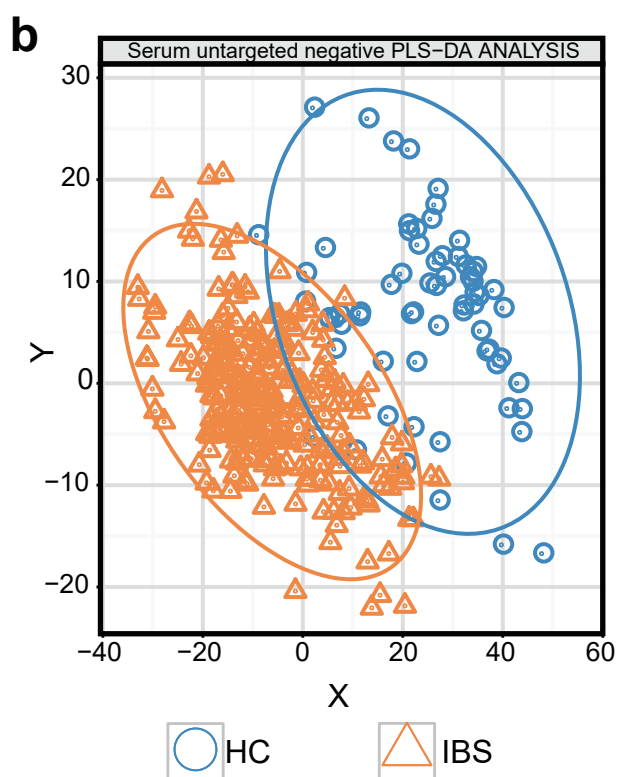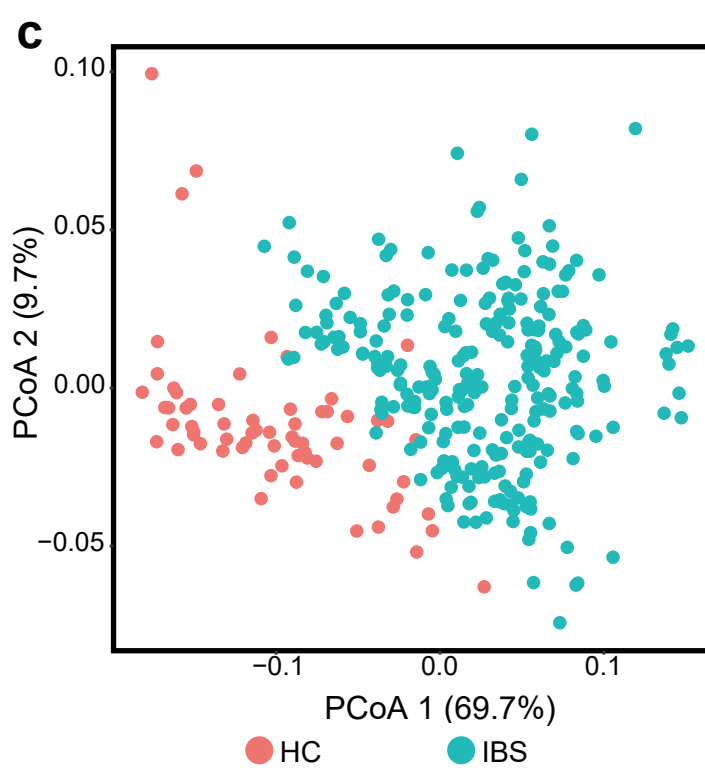

Supplement: Supplementary file 6 — Supplementary Figure 5 [file 41396_2021_1123_MOESM6_ESM.pdf]

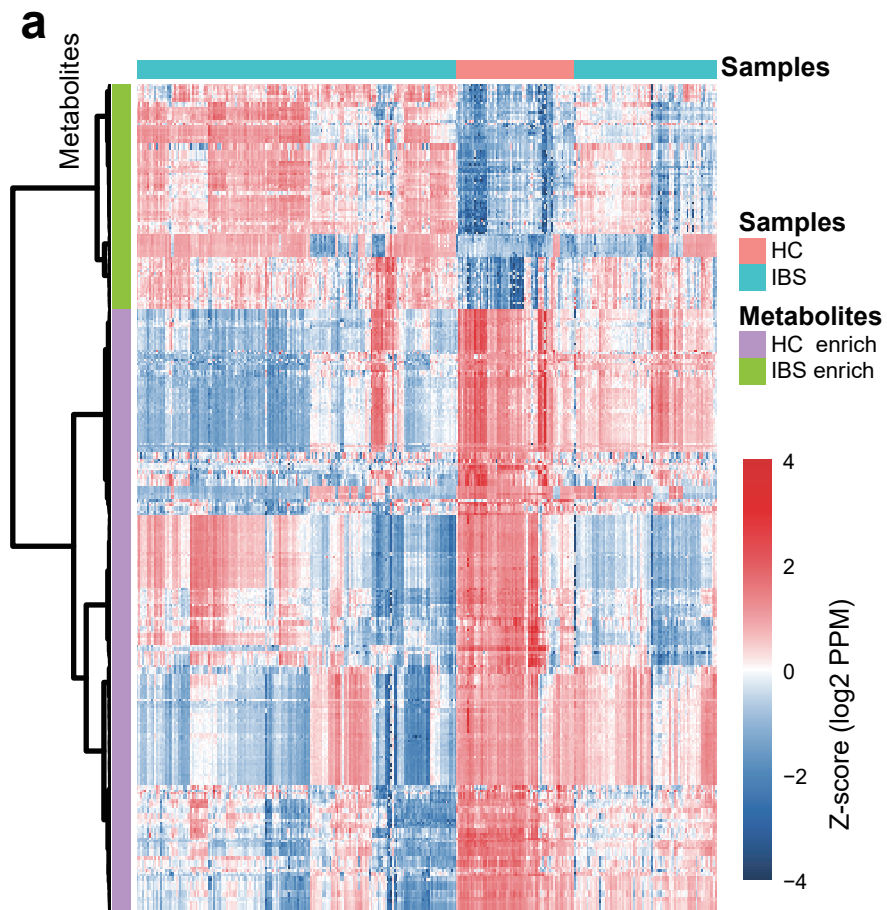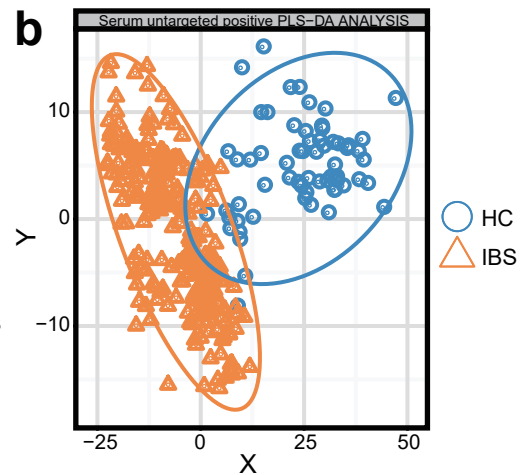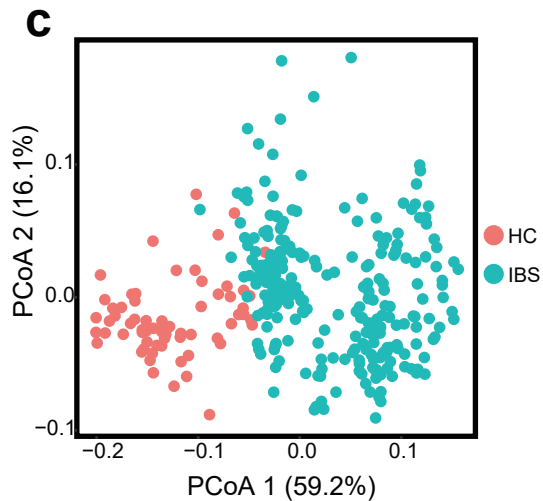

Supplement: Supplementary file 7 — Supplementary Figure 6 [file 41396_2021_1123_MOESM7_ESM.pdf]

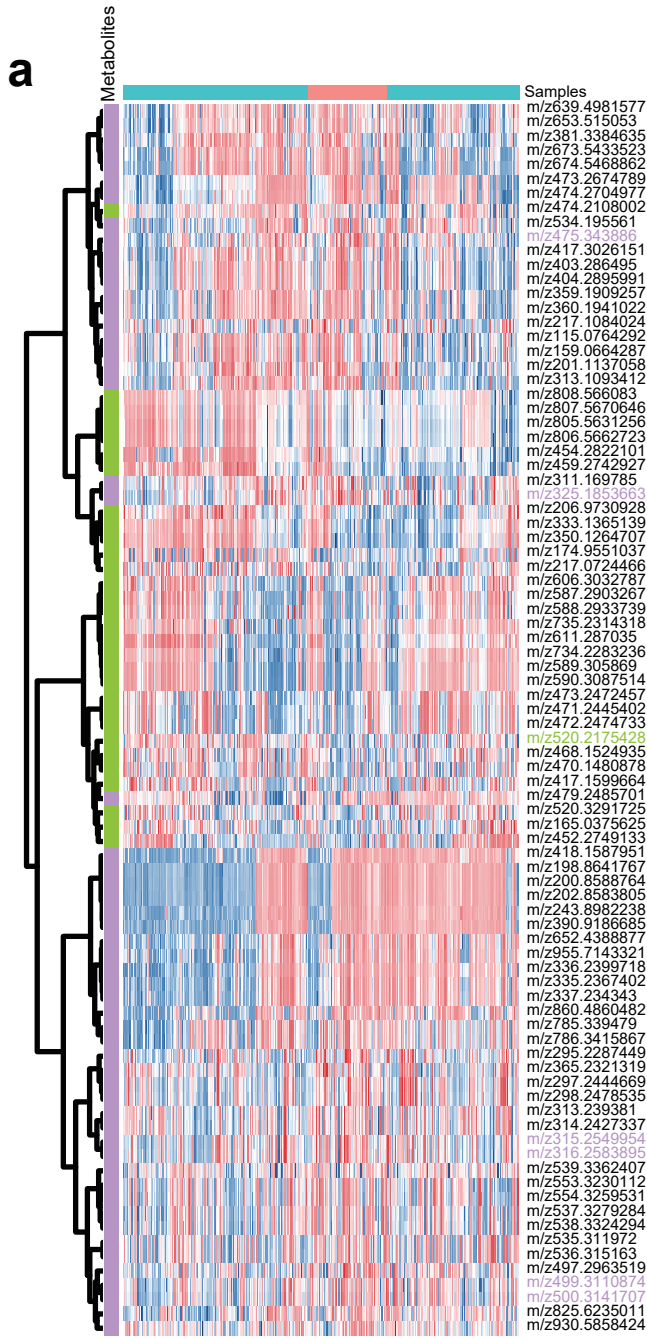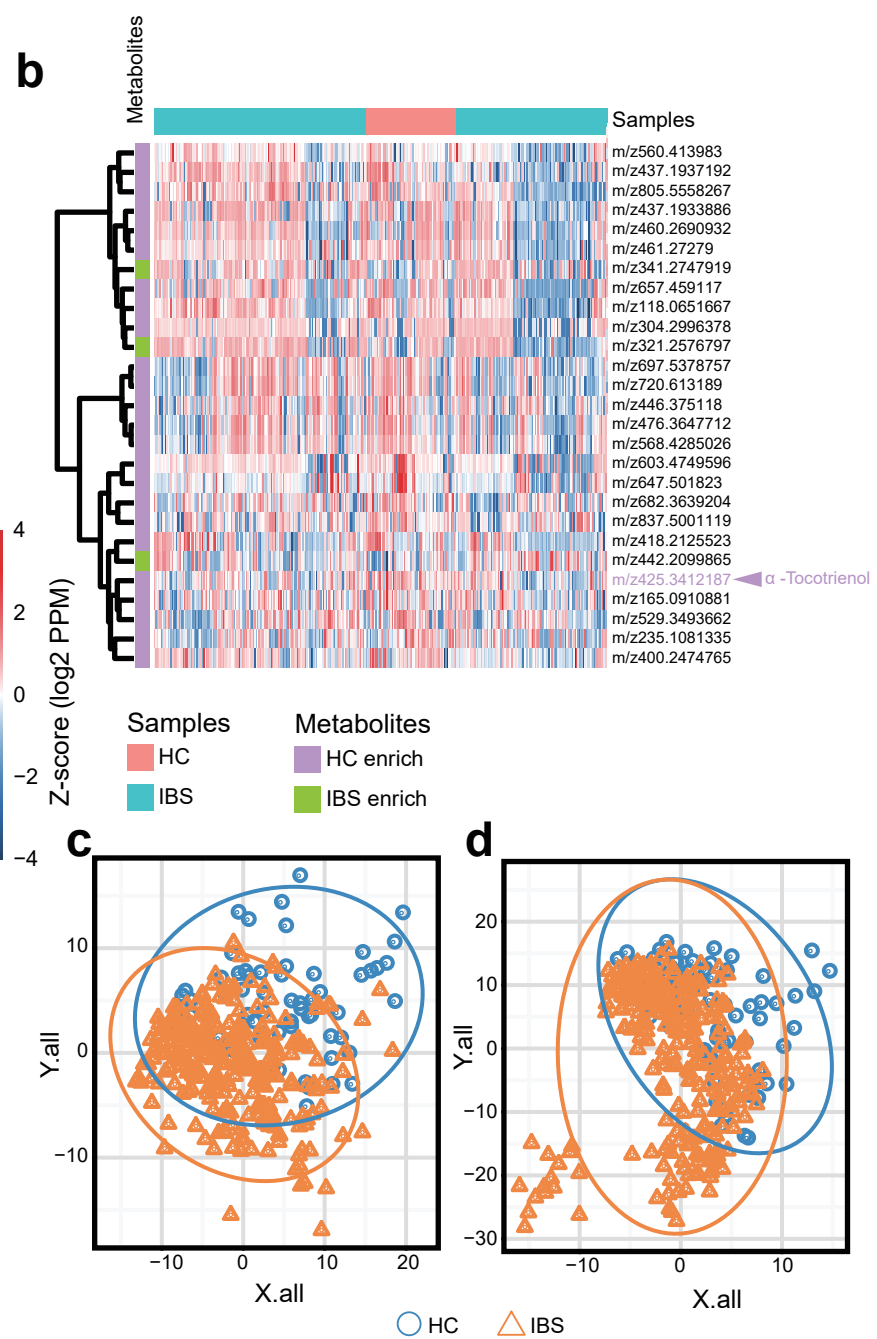

Supplement: Supplementary file 8 — Supplementary Figure 7 [file 41396_2021_1123_MOESM8_ESM.pdf]

**a**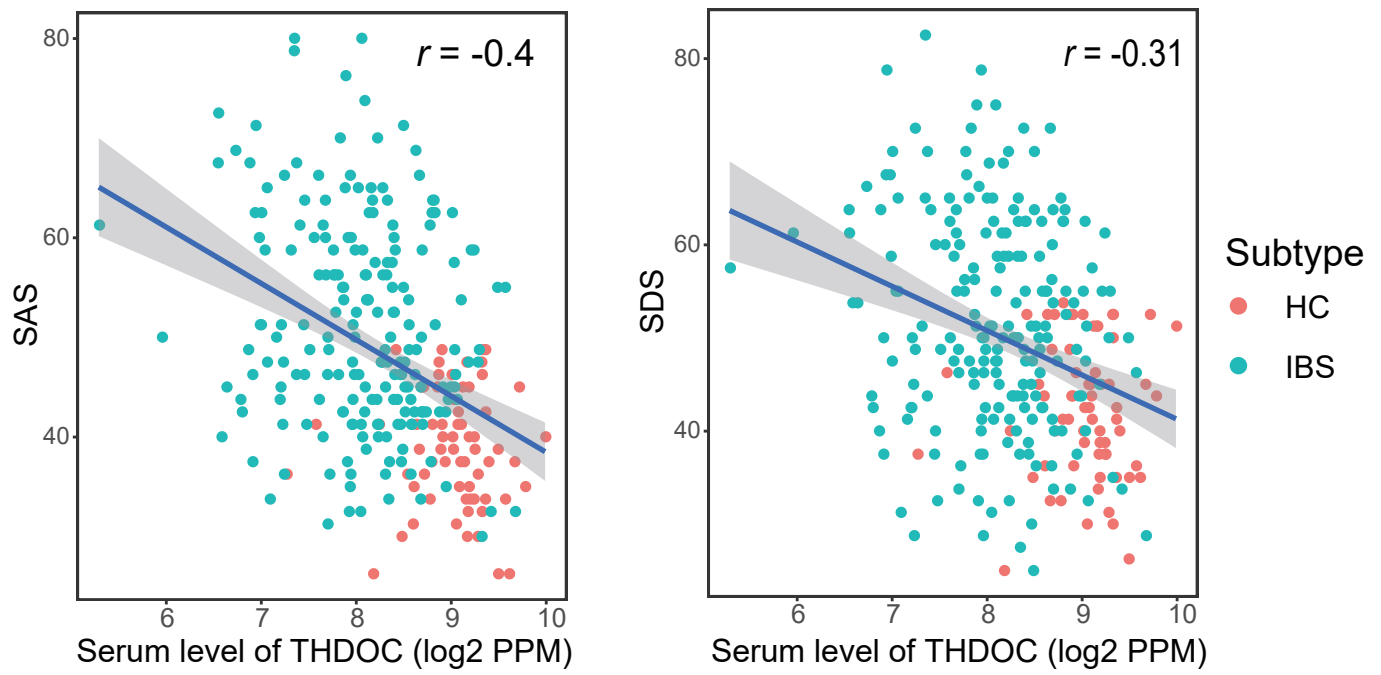**b**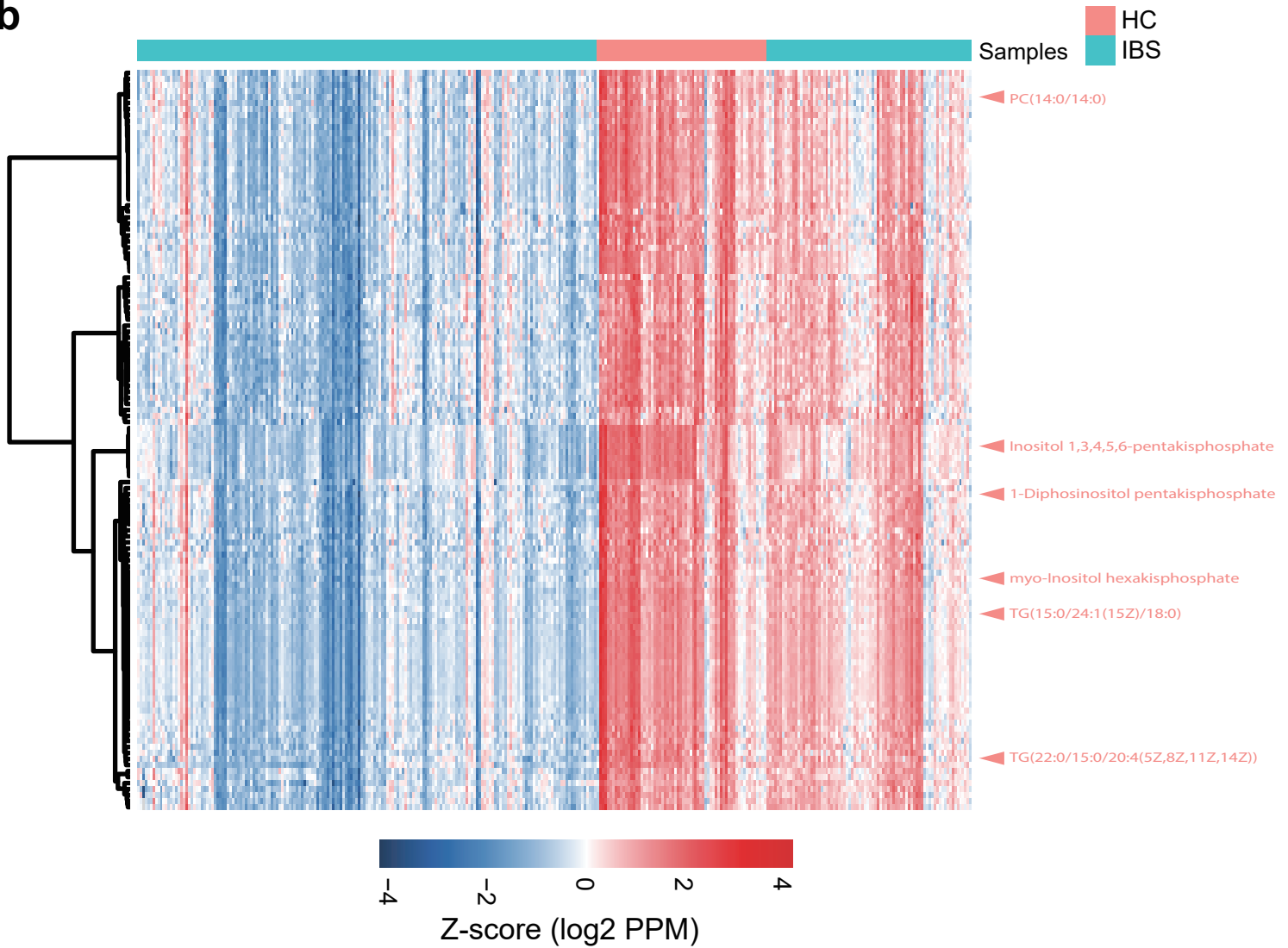

Supplement: Supplementary file 9 — Supplementary Figure 8 [file 41396_2021_1123_MOESM9_ESM.pdf]

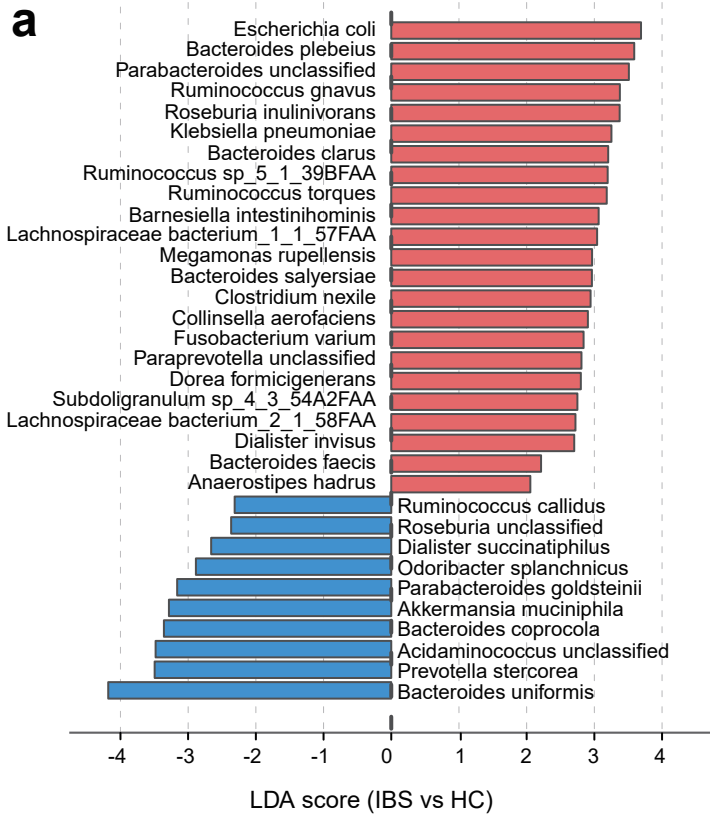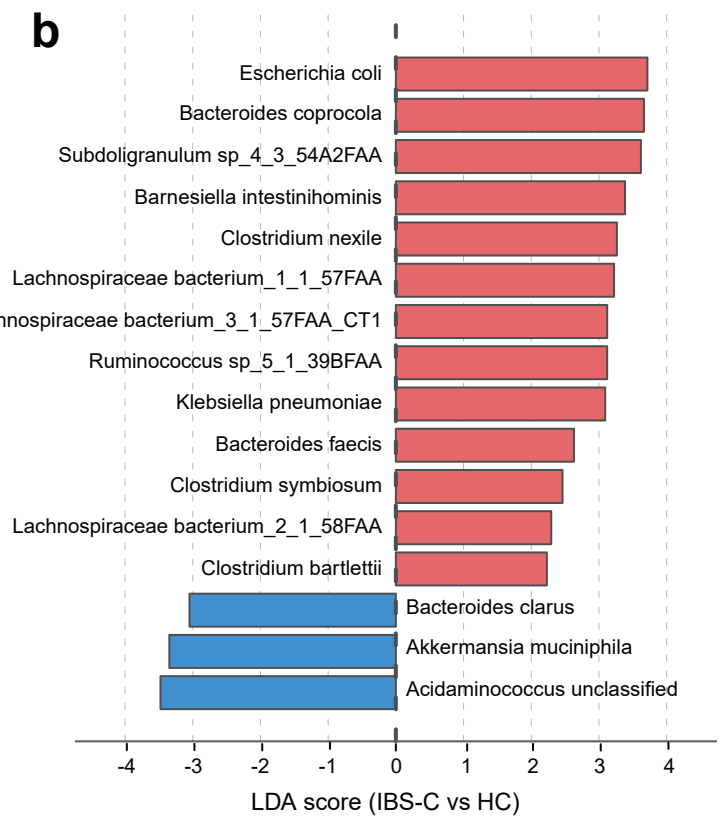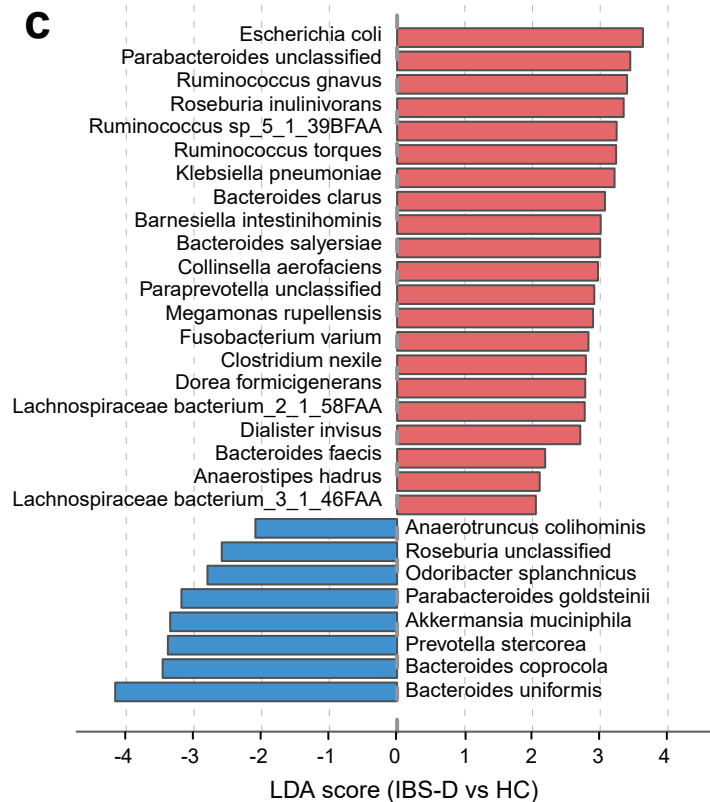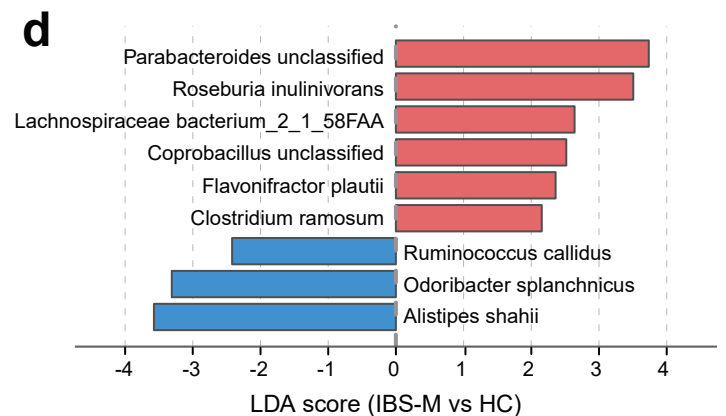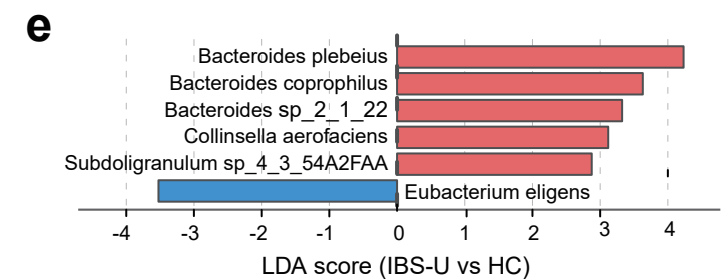

enriched in IBS group

enriched in HC group

Supplement: Supplementary file 10 — Supplementary Figure 9 [file 41396_2021_1123_MOESM10_ESM.pdf]

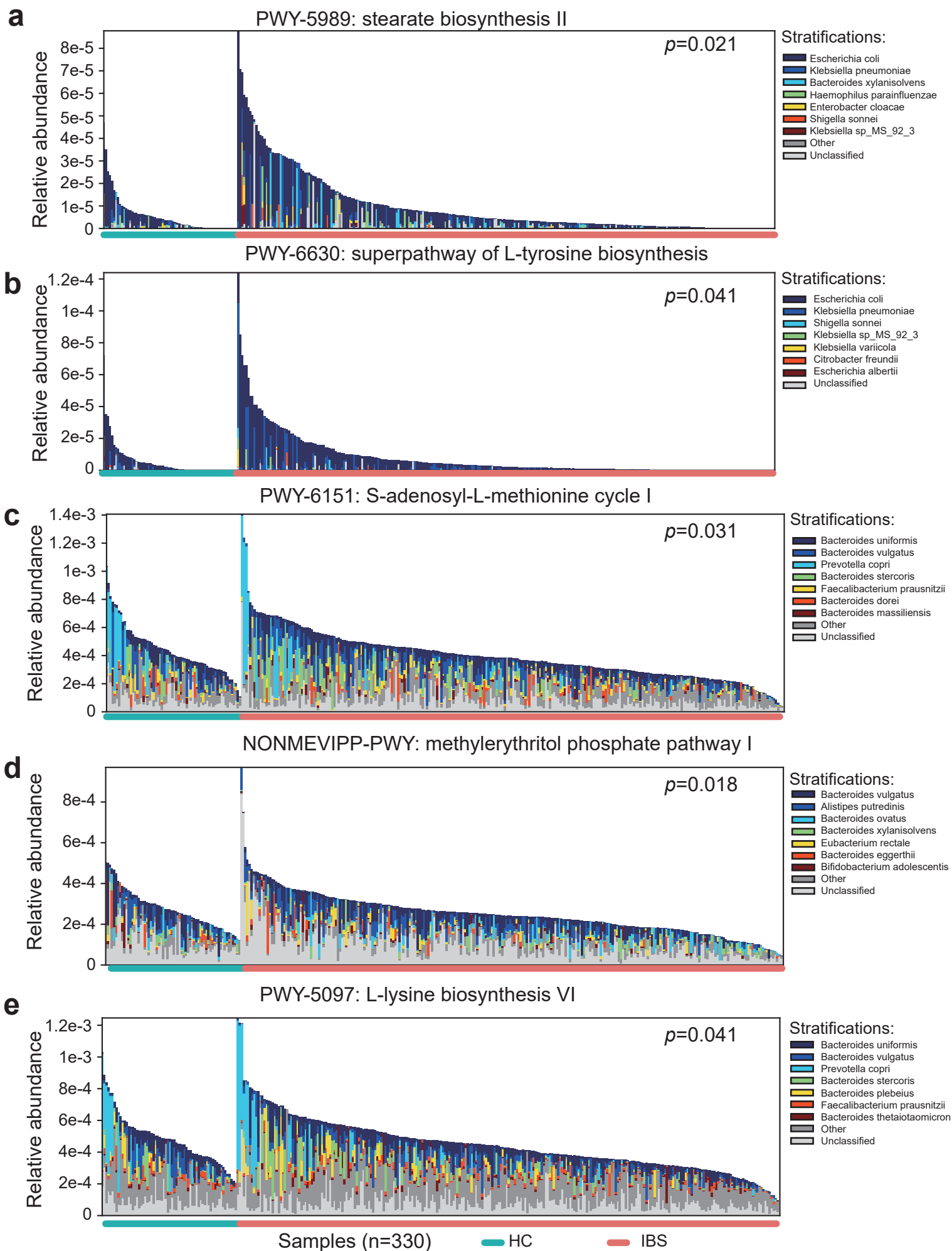

Supplement: Supplementary file 11 — Supplementary Figure 10 [file 41396_2021_1123_MOESM11_ESM.pdf]

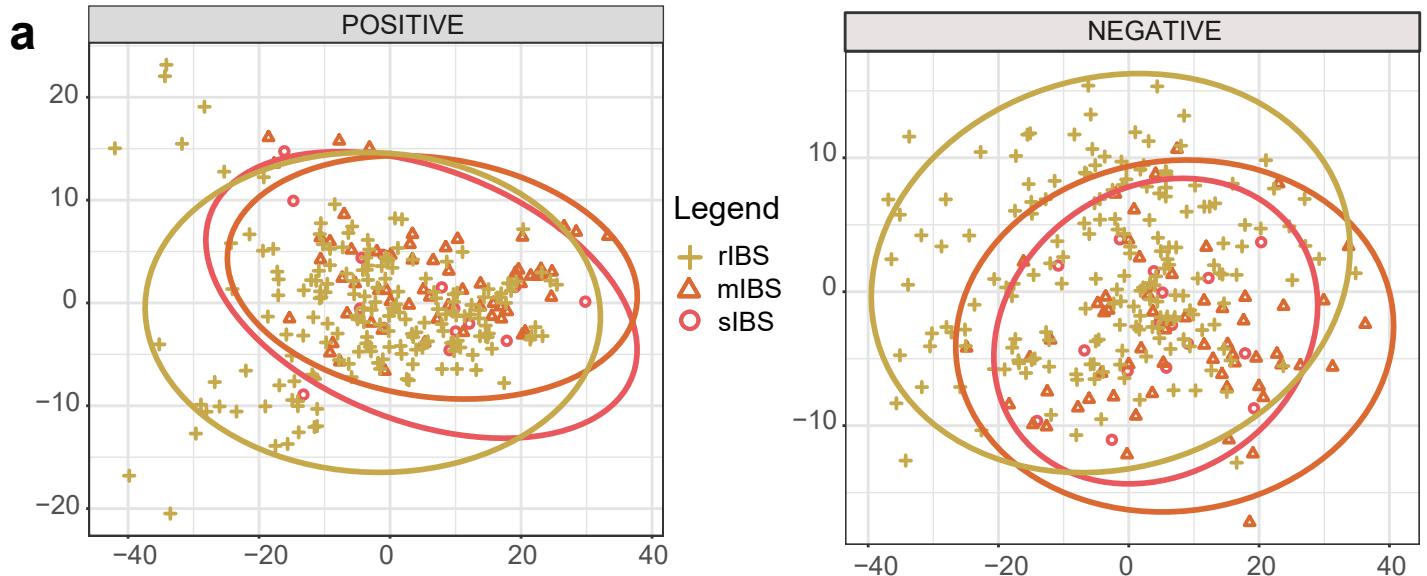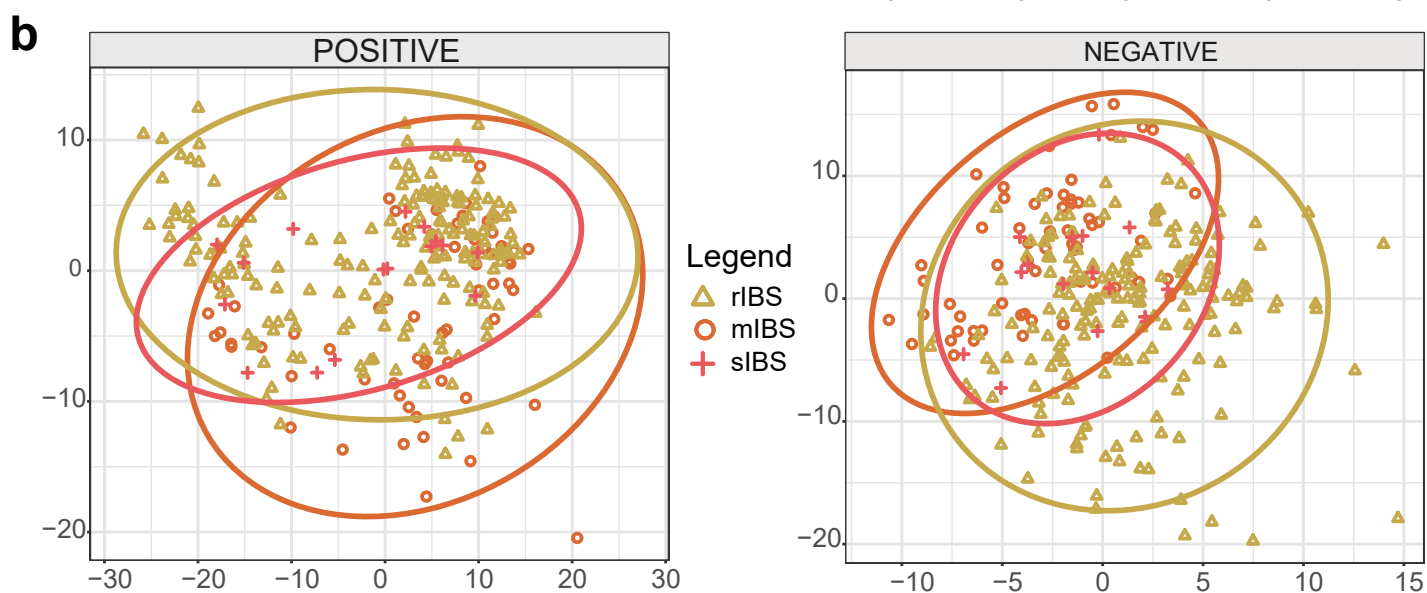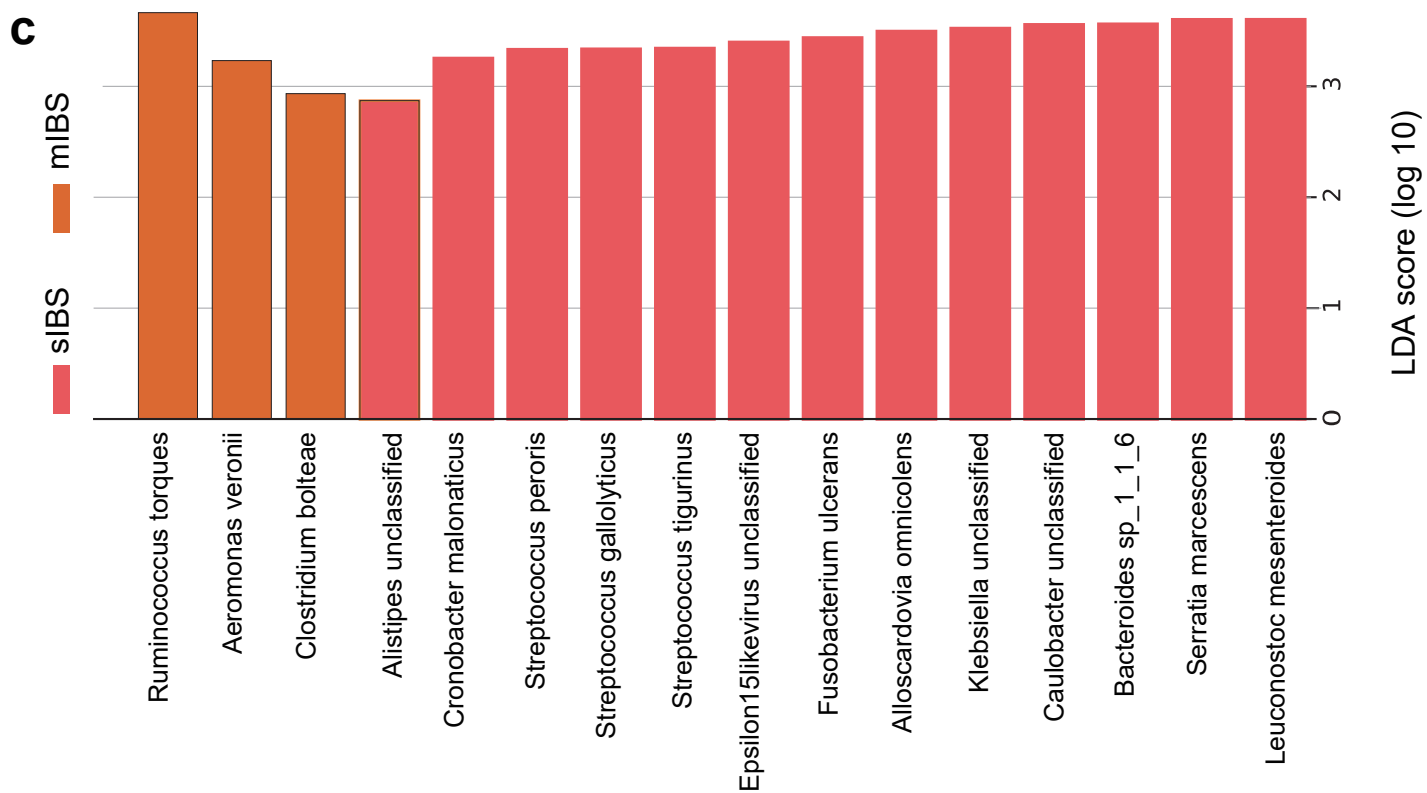

Supplement: Supplementary file 12 — Supplementary Figure 11 [file 41396_2021_1123_MOESM12_ESM.pdf]

**a**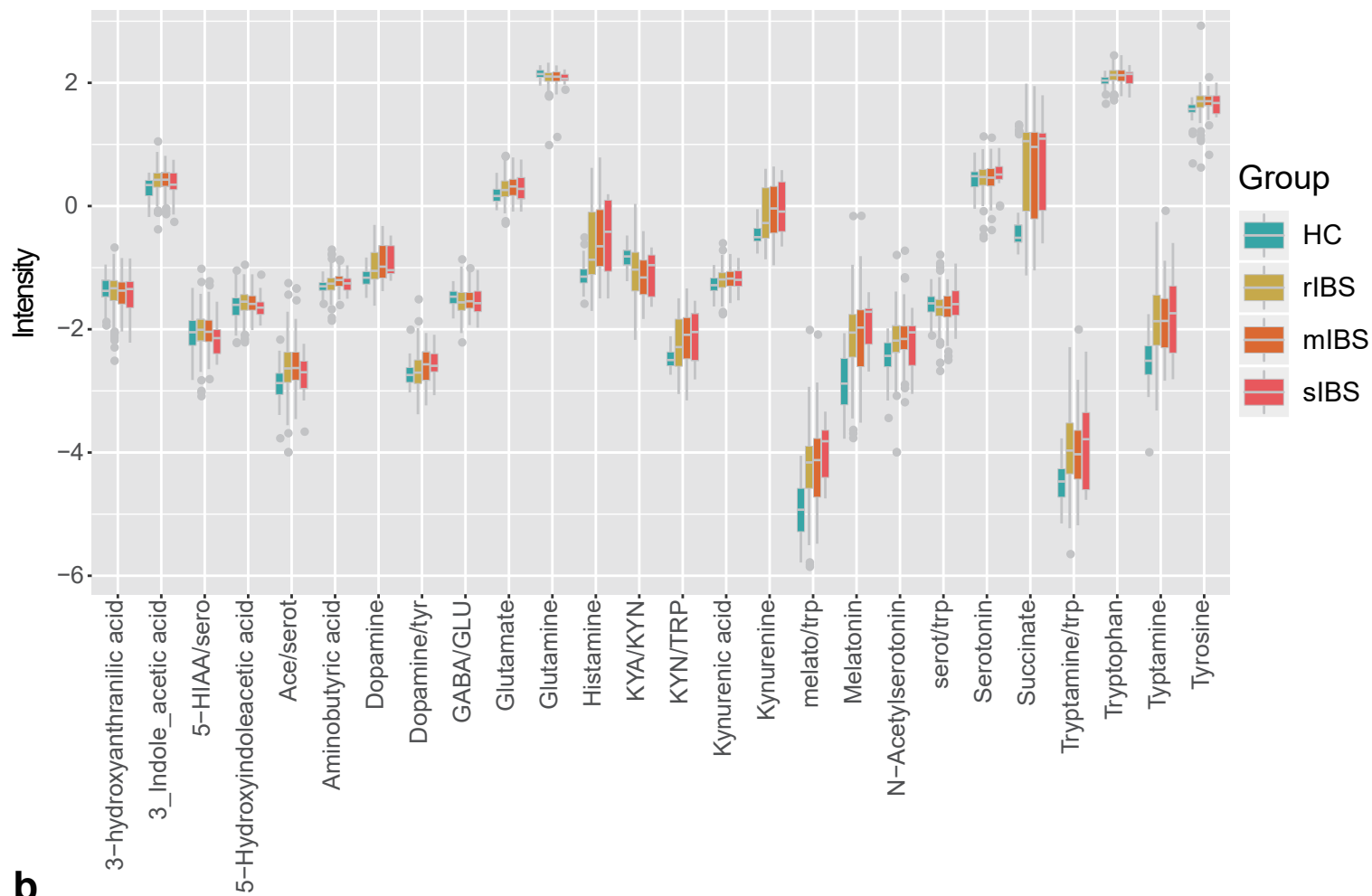**b**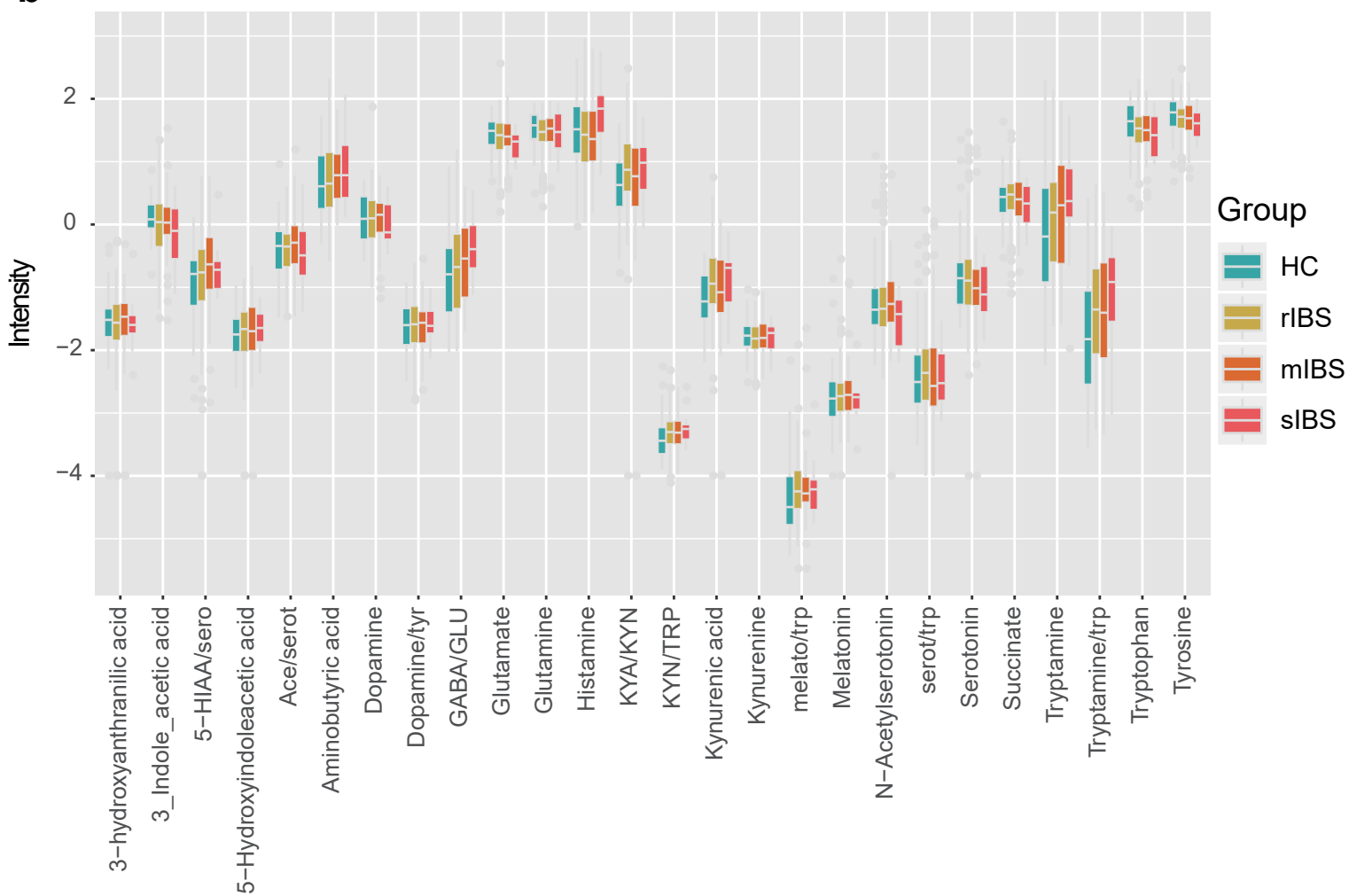

Supplement: Supplementary file 13 — Supplementary Figure 12 [file 41396_2021_1123_MOESM13_ESM.pdf]

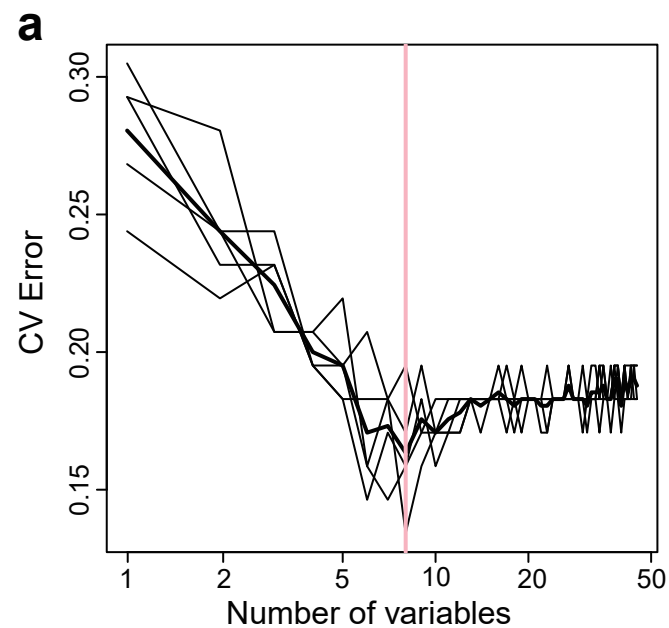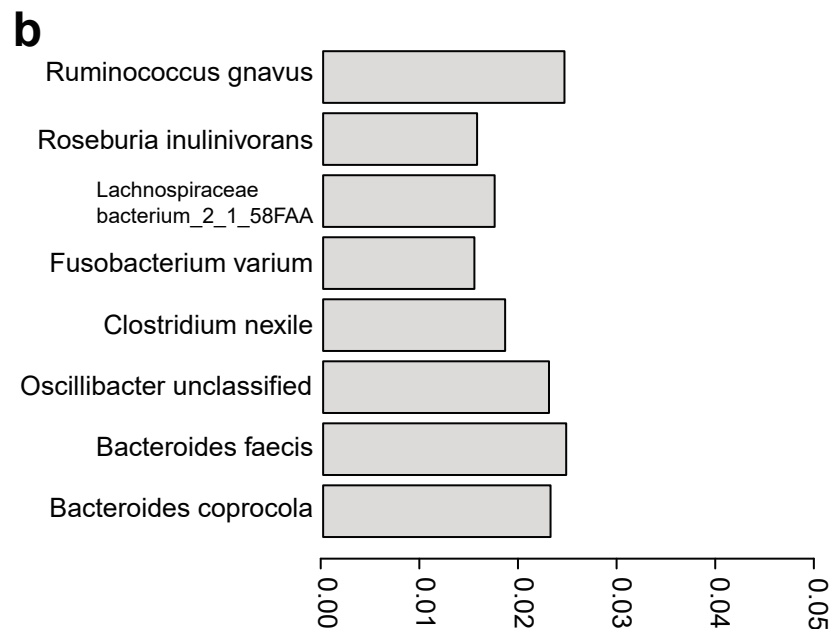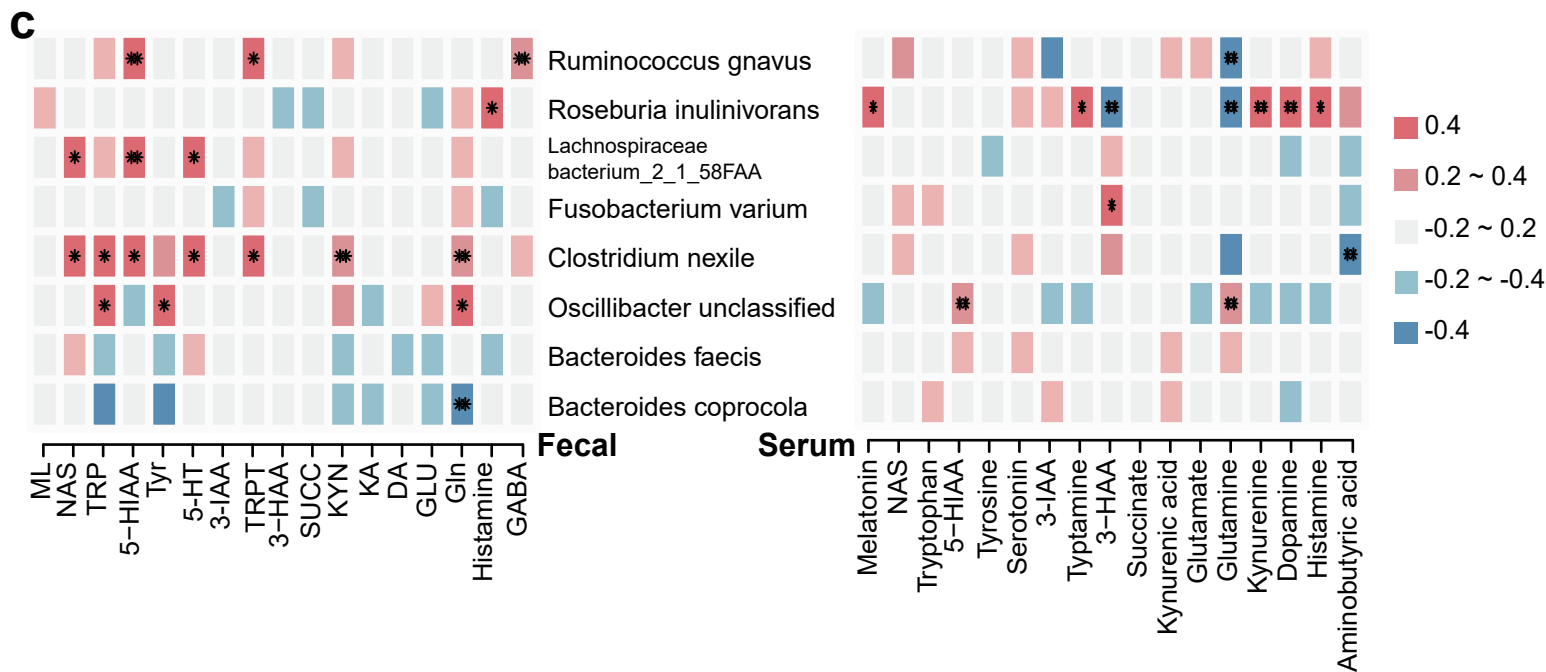

Supplement: Supplementary file 14 — Supplementary Figure 13 [file 41396_2021_1123_MOESM14_ESM.pdf]

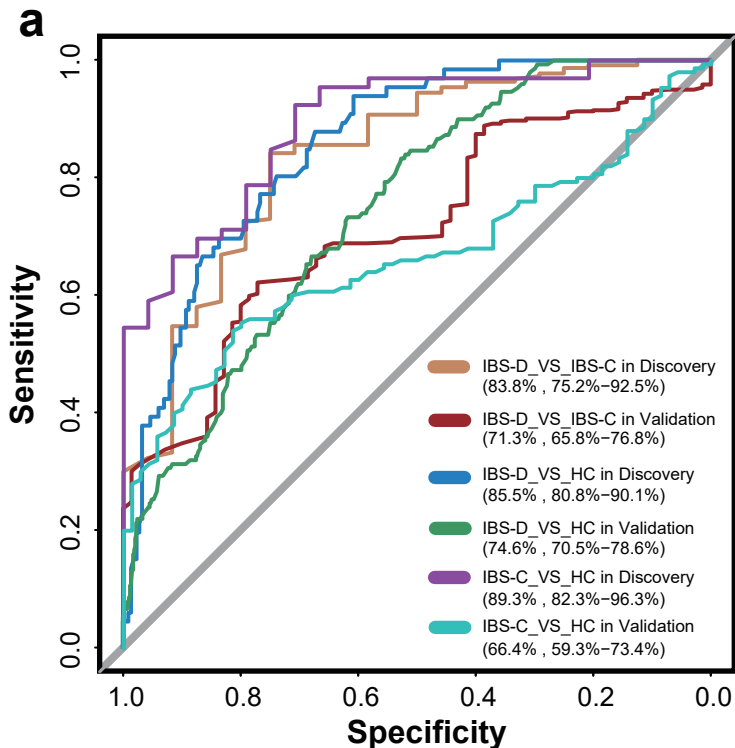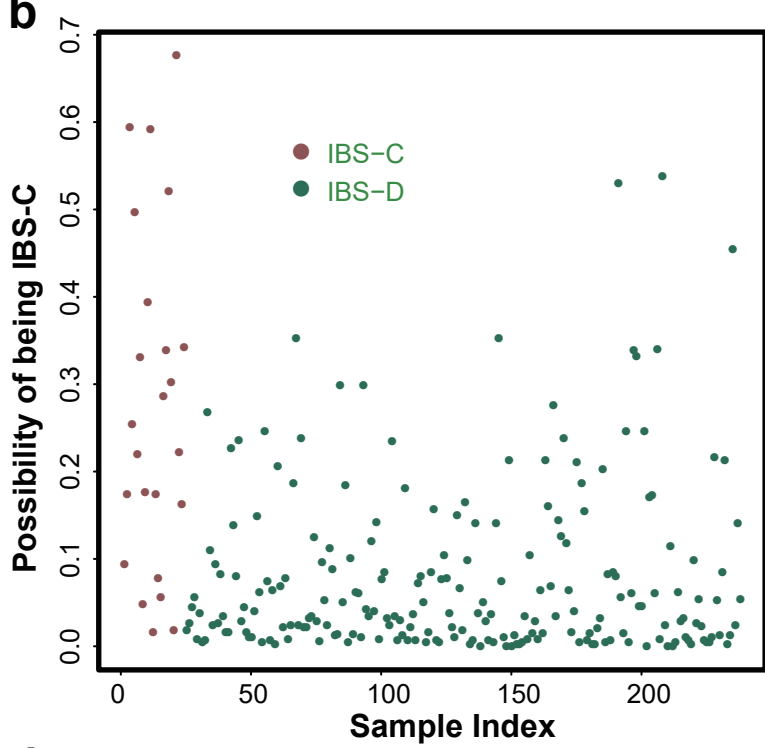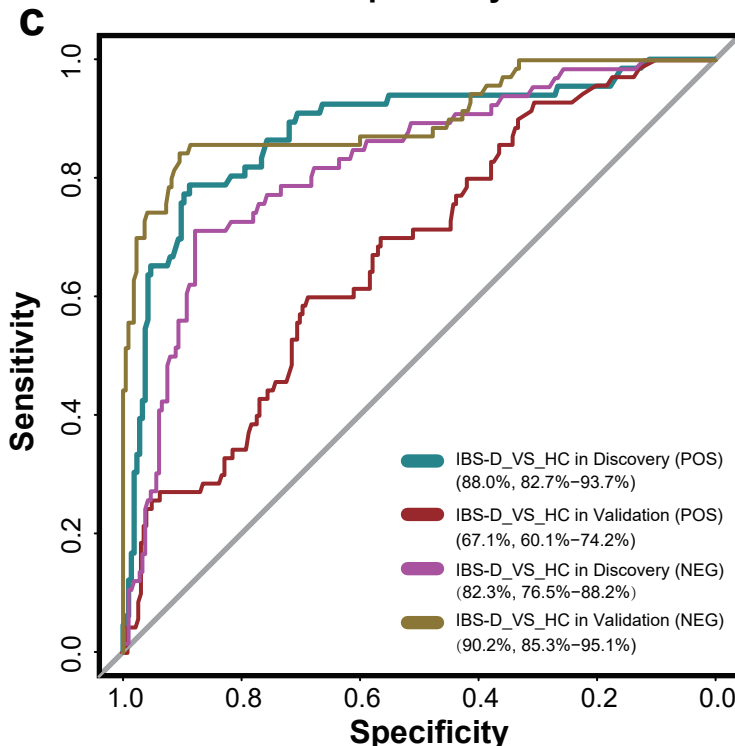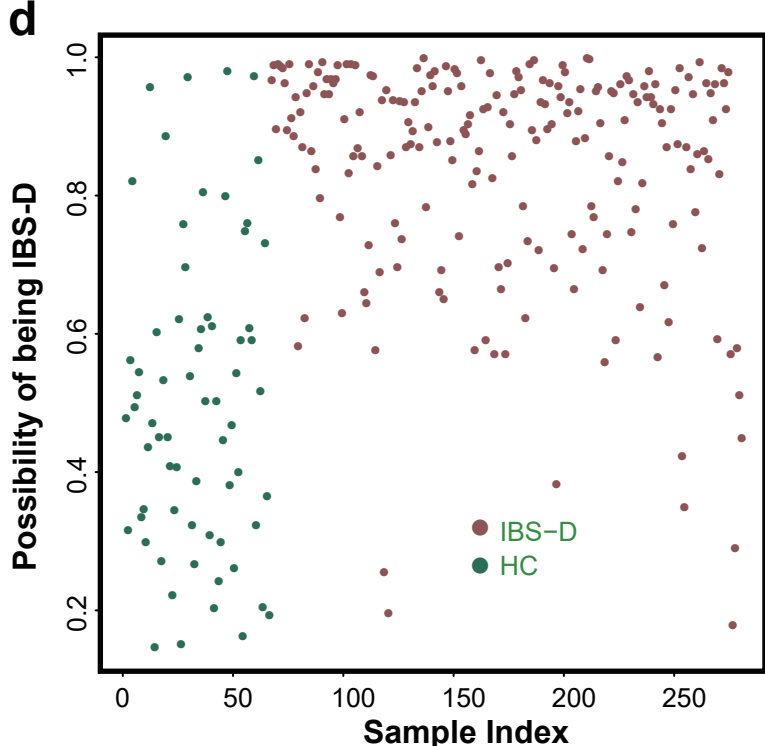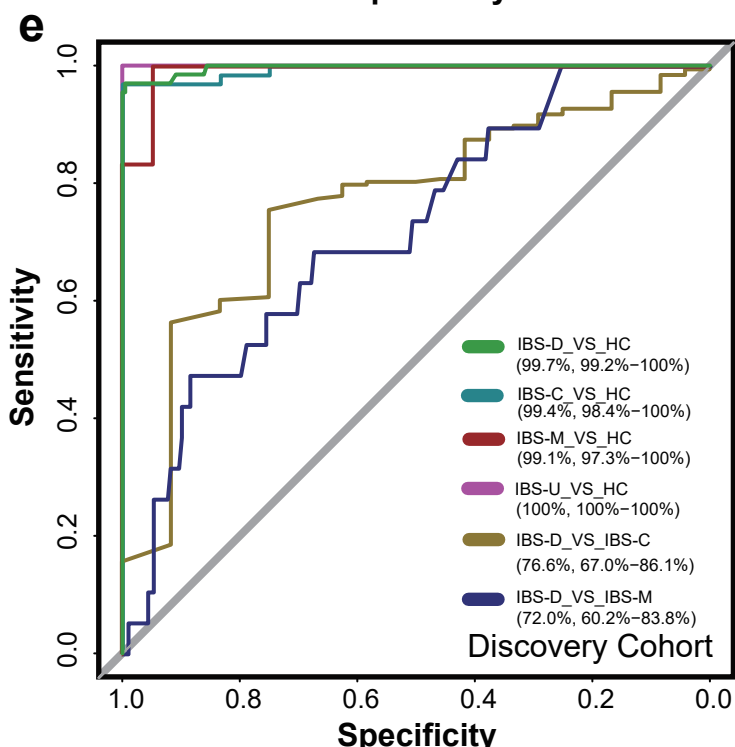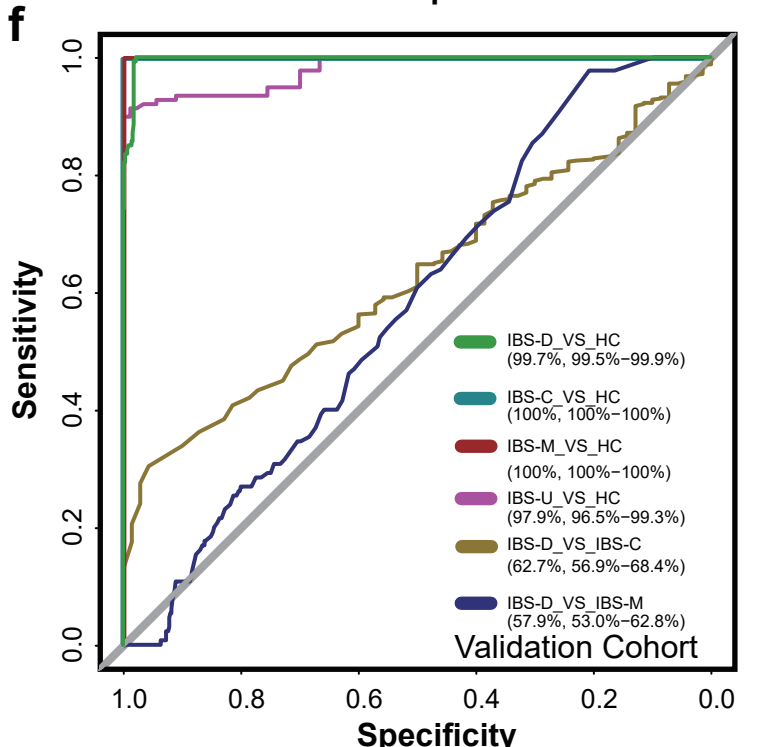

Supplement: Supplementary file 15 — Supplementary Figure 14 [file 41396_2021_1123_MOESM15_ESM.pdf]

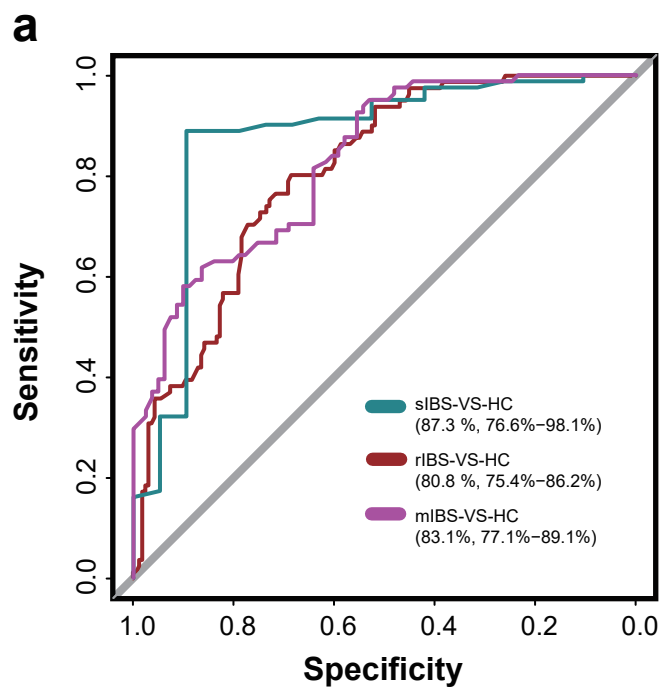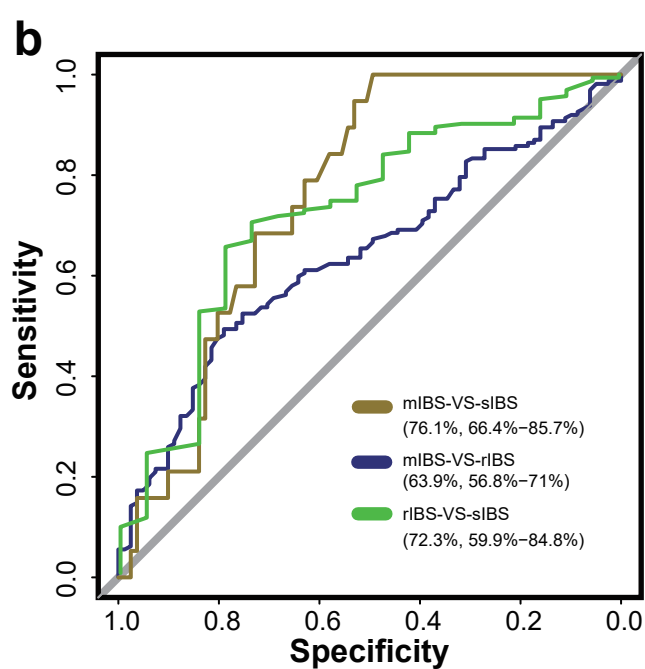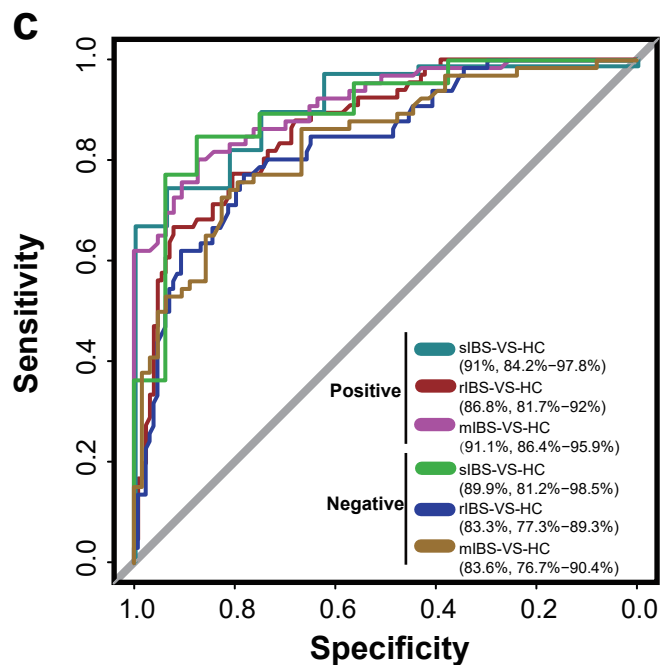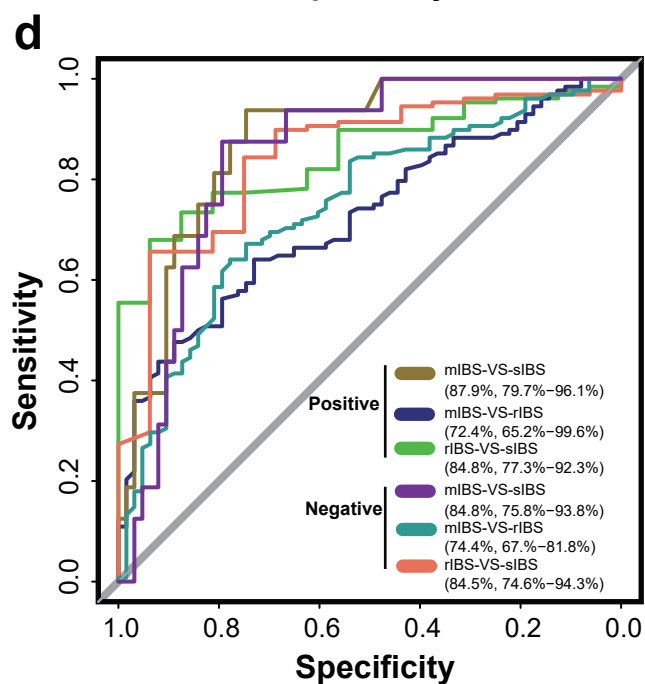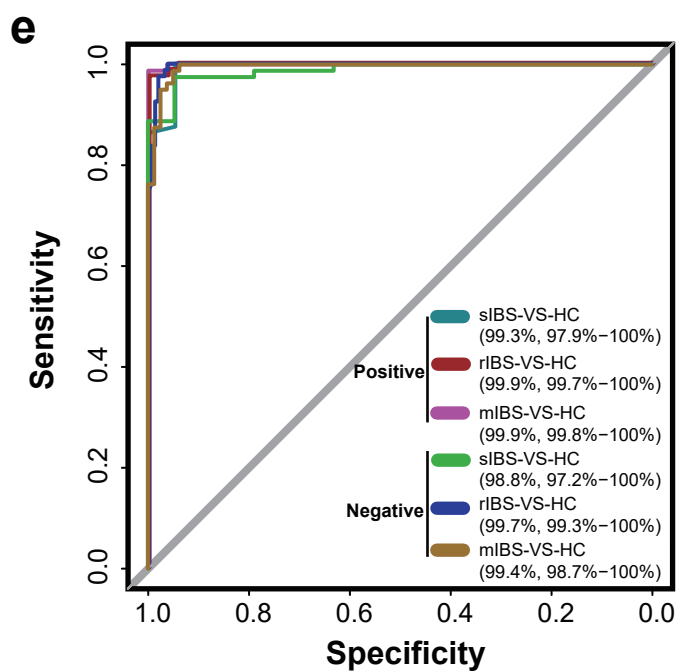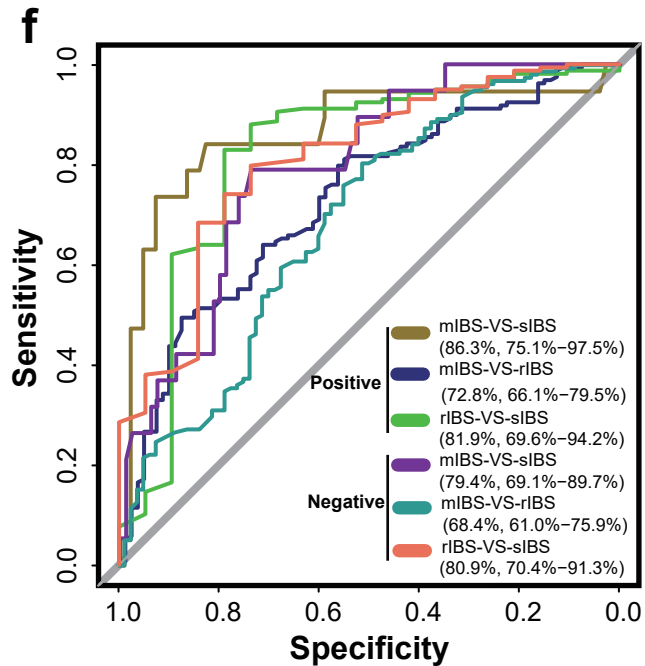

Supplement: Supplementary file 16 — Supplementary Figure 15 [file 41396_2021_1123_MOESM16_ESM.pdf]
